# Taurodontism, variations in tooth number, and misshapened crowns in *Wnt10a* null mice and human kindreds

**DOI:** 10.1002/mgg3.111

**Published:** 2014-09-15

**Authors:** Jie Yang, Shih-Kai Wang, Murim Choi, Bryan M Reid, Yuanyuan Hu, Yuan-Ling Lee, Curtis R Herzog, Hera Kim-Berman, Moses Lee, Paul J Benke, K C Kent Lloyd, James P Simmer, Jan C-C Hu

**Affiliations:** 1 Department of Pediatric Dentistry, School and Hospital of Stomatology, Peking University22 South Avenue Zhongguancun Haidian District, Beijing, 100081, China; 2 Department of Biologic and Materials Sciences, University of Michigan School of Dentistry1210 Eisenhower Place, Ann Arbor, Michigan, 48108; 3 Department of Biomedical Sciences, College of Medicine, Seoul National University275-1 Yongon-dong, Chongno-gu, Seoul, 110-768, Korea; 4 Department of Genetics, Howard Hughes Medical Institute, Yale University School of Medicine333 Cedar Street, New Haven, Connecticut, 06520; 5 Graduate Institute of Clinical Dentistry, National Taiwan UniversityNo. 1 Chang-Te Street, Taipei, 10048, Taiwan, China; 6 Department of Orthodontics and Pediatric Dentistry, University of Michigan School of Dentistry, 1011 N. UniversityAnn Arbor, Michigan, 48109-1078; 7 Department of Medical Genetics, Joe DiMaggio Children's Hospital1150 N. 35th Avenue, Suite 490, Hollywood, Florida, 33021; 8 Mouse Biology Program (MBP), University of California2795 Second Street, Suite 400, Davis, California, 95618

**Keywords:** Familial tooth agenesis, hypodontia, oligodontia, taurodontism

## Abstract

WNT10A is a signaling molecule involved in tooth development, and *WNT10A* defects are associated with tooth agenesis. We characterized *Wnt10a* null mice generated by the knockout mouse project (KOMP) and six families with *WNT10A* mutations, including a novel p.Arg104Cys defect, in the absence of *EDA*,*EDAR*, or *EDARADD* variations. *Wnt10a* null mice exhibited supernumerary mandibular fourth molars, and smaller molars with abnormal cusp patterning and root taurodontism. *Wnt10a*^*−/−*^ incisors showed distinctive apical–lingual wedge-shaped defects. These findings spurred us to closely examine the dental phenotypes of our *WNT10A* families. *WNT10A* heterozygotes exhibited molar root taurodontism and mild tooth agenesis (with incomplete penetrance) in their permanent dentitions. Individuals with two defective *WNT10A* alleles showed severe tooth agenesis and had fewer cusps on their molars. The misshapened molar crowns and roots were consistent with the *Wnt10a* null phenotype and were not previously associated with *WNT10A* defects. The missing teeth contrasted with the presence of supplemental teeth in the *Wnt10a* null mice and demonstrated mammalian species differences in the roles of Wnt signaling in early tooth development. We conclude that molar crown and root dysmorphologies are caused by *WNT10A* defects and that the severity of the tooth agenesis correlates with the number of defective *WNT10A* alleles.

## Introduction

Early tooth development progresses through serial epithelial–mesenchymal interactions (Lumsden 1988; Thesleff 2006). Expression of critical morphogens in specific epithelial domains of the first branchial arch defines odontogenic fields and initiates tooth formation. The subsequent epithelial invagination and convolution establish the distinct anatomical and functional parts of the tooth and determine the basic shape of the tooth crown. Specifically, the enamel knot, an epithelial structure serving as a signaling center, controls this morphogenesis process (Jernvall et al. 1994). Many genes and signaling pathways functioning at the enamel knot have been shown to be critical for cusp patterning of the tooth crown, such as Shh, Bmp, and Wnt signaling (Lan et al. 2014). On the other hand, root development begins after crown morphogenesis is complete. The Herwig's epithelial root sheath (HERS) derives from enamel organ epithelium and guides root morphogenesis. Activation of several signaling pathways, such as Wnt and Tgf-*β* signaling, around HERS have been demonstrated to play significant roles in root formation (Kumakami-Sakano et al. 2014). Aberrations in genes critical to these sequential developmental processes lead to failed tooth development, abnormal crown patterning, or altered root morphogenesis (Cobourne and Sharpe 2013).

*WNT10A* (Wingless-type MMTV integration site family, member 10A; OMIM * 606268) is a member of the *WNT* gene family, which consists of structurally related genes encoding secreted signaling molecules critical for many organogenesis processes (Liu and Millar 2010; Lan et al. 2014). Mouse *Wnt10a* was first cloned and shown to be widely expressed in various tissues of the adult and embryo (Wang and Shackleford 1996). Delineating the role of epithelial signaling molecules during early tooth development, Dassule and McMahon showed that *Wnt10a* is expressed first in the dental epithelial thickening (dental placode) and later in the primary and secondary enamel knots, so *Wnt10a* potentially functions during tooth initiation and crown patterning (Dassule and McMahon 1998). Yamashiro et al. (2007) demonstrated that *Wnt10a* is expressed later during tooth development, by secretory odontoblasts lining dentin and differentiating odontoblasts around HERS, so *Wnt10a* also potentially functions during dentin and root development.

The significance of *WNT10A* in organogenesis was not recognized until it was discovered that *WNT10A* mutations can cause Odonto-onycho-dermal Dysplasia (OODD; MIM#257980), a rare autosomal recessive syndrome characterized by sparse hair, severe tooth agenesis, smooth tongue with marked reduction of fungiform and filiform papillae, onychodysplasia, keratoderma, and hyperhidrosis (Adaimy et al. 2007). Bohring et al. (2009) reported that about half of the heterozygous carriers in OODD families exhibit mainly tooth and nail abnormalities without other apparent ectodermal dysplasia phenotypes, which established *WNT10A* as a candidate gene for nonsyndromic tooth agenesis. Already more than 60 different mutations affecting one or both *WNT10A* alleles have been identified in persons with nonsyndromic tooth agenesis in a variety of ethnic populations (Table S1), which indicate there is a high variability in disease severity (number of missing teeth) even in cases with identical genotypes. It has only recently become apparent, however, that the severity of the tooth agenesis is increased if both *WNT10A* alleles are defective or if a *WNT10A* defect is combined with defects in *ectodysplasin A* (EDA; OMIM *300451), *ectodysplasin A receptor* (*EDAR*; OMIM * 604095), or *EDAR-associated death domain* (*EDARADD*; OMIM * 606603) (Arte et al. 2013; He et al. 2013).

In this study we characterize the dental phenotypes of *Wnt10a* null mice and in six families with *WNT10A*-associated tooth agenesis, in the absence of any contributory defects in *EDA*,*EDAR*, or *EDARADD*. We describe variations in tooth number and altered tooth crown morphology and root taurodontism in both *Wnt10a* null mice and in human patients, demonstrating that WNT10A plays significant roles in determining tooth number and in the patterning and morphogenesis of tooth crowns and roots. Our findings support the need for future *WNT10A* studies to also identify potential disease-causing sequence variants in interacting genes and to characterize crown and root dysmorphologies, given the ambiguities and discrepancies in the literature concerning the severity of tooth agenesis and dental phenotypes associated with *WNT10A* mutations.

## Materials and Methods

The human study protocol and consents were reviewed and approved by the IRB Committee at the National Taiwan University Hospital, Taipei, Taiwan and the Institutional Review Board at the University of Michigan. Study participants signed appropriate written consents after explanation and discussion of their contents. The care, use, and disposition of all mice used in this study were reviewed and approved by the Institutional Animal Care and Use Committee of the University of California Davis.

### Generation of *Wnt10a* knockout mice

*Wnt10a* null mice were generated by the Mouse Biology Program using gene-targeted embryonic stem (ES) cells produced by Velocigene, Regeneron Pharmaceuticals, Inc (Tarrytown, NY) for the NIH Knockout Mouse Project (KOMP). The knockout deleted 11,515 bp (74,838,748 to 74,850,262 of mouse chromosome 1, genome build 37) that included the entire *Wnt10a* coding region. The knockout construct replaced the *Wnt10a* gene in ES cells (C57BL/6NTac background) with a ZEN-Ub1 cassette that introduced a bacterial *lacZ* code at the natural *Wnt10a* translation initiation codon (in exon 1) and a downstream neomycin phosphotransferase gene (*neo*^r^) driven by the human ubiquitin C gene promoter (hUBCpro). The neomycin resistance gene was bracketed by a locus of X-over P1 sequence from bacteriophage P1 (loxP) sequences for convenient removal of the *neo*^r^ selection code. Successful knockout was determined by PCR genotyping (http://www.velocigene.com/komp/detail/14810). Three archived *Wnt10a* null mouse heads (1316, 1317, and 1329) and one archived wild-type head (1331) from 16-week-old mice were provided to us from KOMP for analysis.

### Radiography and microscopic photography

The mandibles were removed from the four 16-week-old archived heads provided by KOMP: *Wnt10a*^+/+^ specimen 1331 and *Wnt10a*^−/−^ specimens 1316, 1317, 1329, and sliced through the mental symphysis with a razor blade to generate hemimandibles. These were carefully dissected of soft tissues under a stereoscopic microscope using tissue forceps and a spoon excavator. The cleaned hemimandibles were radiographed using a Faxitron X-ray cabinet model MX-20 (Faxitron X-ray Corp., Wheeling, IL) operating at 32 kV for 120 sec and photographed using a Nikon SMZ1000 dissection microscope equipped with a Nikon digital camera DXM1200 (Mager Scientific, Dexter, MI).

### Backscatter scanning electron microscopy

Molars were extracted from the left mandibles and debrided by soaking in 1% sodium hypochlorite for 10 min followed by ultrasound for 4 min. The right hemimandibles and molars were dehydrated by gradient acetone (30, 50, 70, 80, 90, and 100%), mounted on 1″ aluminum sample stubs, and coated with carbon. Backscatter scanning electron microscopy (SEM) evaluation was performed using a Hitachi S-3000N variable pressure scanning electron microscope (Hitachi High Technologies America, Schaumburg, IL) under backscatter mode at 15 kV and 20 Pa pressure at the University of Michigan Microscopy and Image analysis Laboratory (Ann Arbor, MI). Images were obtained at ×30, ×200, and ×600 magnification.

### Microcomputed tomography

All eight hemimandibles were embedded in 1% agarose and placed in a 19-mm-diameter tube and scanned over the entire length of the mandible using a microCT system (*μ*CT100 Scanco Medical, Bassersdorf, Switzerland) at the University of Michigan School of Dentistry Microcomputed tomography (*μ*CT) core. Scan settings were as follows: voxel size 8 *μ*m, 70 kVp, 114 *μ*A, 0.5 mm aluminum filter, and integration time of 500 msec. The system was calibrated with a manufacturer-provided phantom consisting of 0, 100, 200, 400, 800, and 1200 mg HA/cm^3^ phantom rods embedded in resin. The data were analyzed using the *μ*CT Evaluation Program V6.5-1. For the 3D molar images, the outside contour of each molar and incisor was outlined by marking the border of the molar/incisor on each scanning section. 3D images were generated within the contour-defined area. Because mature enamel is more dense than dentin and dentin is more dense than pulp, the enamel, dentin, and pulp portions of the molars were isolated using density threshold ranges for the purpose of measuring volume and density. The threshold range for measuring the entire tooth was 0–1000, for pulp was 0–260, for dentin was 260–650 (colored gray), and for enamel was 650–1000 (colored white). Enamel, dentin, and pulp volumes were calculated for each tooth using the *μ*CT Evaluation Program V6.5-1 and the volumes within the contour-defined area of each tooth that corresponded to the threshold ranges described above. Virtual sagittal images were generated by cutting through the central plane of the 3D images using the image analysis software.

### Recruitment of subjects

Study participants signed appropriate written consents after an explanation of their contents and after their questions about the study were answered. Minors age 8 or older signed a written assent form after their parent completed a written parental consent for participation of the minor.

### Genomic DNA extraction

Peripheral whole blood (5 mL) or saliva (2 mL) was obtained from recruited individuals and genomic DNA was isolated using the QIAamp DNA Blood Maxi Kit (51194; Qiagen, Valencia, CA) or Saliva DNA Collection, Preservation, and Isolation Kit (RU35700; Norgen Biotek Corporation; Thorold, Canada), respectively. The quality and quantity of the extracted DNA samples were determined by spectrophotometry at OD_260_ and OD_280_.

### Whole-exome analyses

Whole-exome sequencing was conducted at the University of Michigan DNA Sequencing Core and Yale Center for Genome Analysis (West Haven, CT). In the University of Michigan DNA Sequencing Core, genomic DNA (3 *μ*g) was characterized by whole-exome sequencing using an Illumina TruSeq Exome Enrichment system and HiSeq 2000 platform at 75 base paired-end sequencing (San Diego, CA). Sequence output was inspected and aligned against human reference genome hg19, and variants were filtered and annotated using Ingenuity Variant Analysis tool by the University of Michigan Bioinformatics Core. In the Yale Center for Genome Analysis, the exome sequencing and subsequent analysis were modified from a previous report (Choi et al. 2009). Briefly, the genomic DNA was captured with NimblGen v2.0 exome capture reagent (Roche/NimblGen Incorporation; Madison, WI) and sequenced with Illumina HiSeq 2000 for 75 base paired-end reads. Reads were aligned to human reference genome hg19 using ELAND v2. Single-nucleotide variants and short insertions and deletions (indels) were called using SAM tools. The called variants were annotated using an in-house script. The annotated results were first inspected to search for potential disease-causing sequence variations in the known candidate genes for syndromic and nonsyndromic tooth agenesis. In families with multiple exomes being sequenced, the data from each were compared, and the sequence variations were further filtered based upon disease segregation. In some circumstances, exome data from unrelated affected individuals were compared and searched for sequence variations for the same gene. Eventually, potentially disease-causing sequence variations were confirmed in the probands and all participating family members by Sanger sequencing.

### *WNT10A* mutational analyses

The coding exons and intron junctions for *WNT10A*,*EDA*,*EDAR*, and *EDARADD* were amplified by polymerase chain reaction (PCR) using specific oligonucleotide primer pairs (Table S2). The amplification products were purified and characterized by direct DNA sequencing (Sanger sequencing) at the University of Michigan DNA Sequencing Core. The sequencing data were then compared to the human reference sequence, and sequence variants called and evaluated. *WNT10A* c.DNA and genomic changes were numbered with respect to the National Center for Biotechnology Information (NCBI) human *WNT10A* mRNA reference sequence: NM_025216.2 (numbered from the first nucleotide of the *WNT10A* translation initiation codon in exon 1) and genomic reference sequence NG_012179.1 (numbered from the first nucleotide of the reference sequence).

## Results

### Supernumerary molars in *Wnt10a* null mice

**Figure 1:**
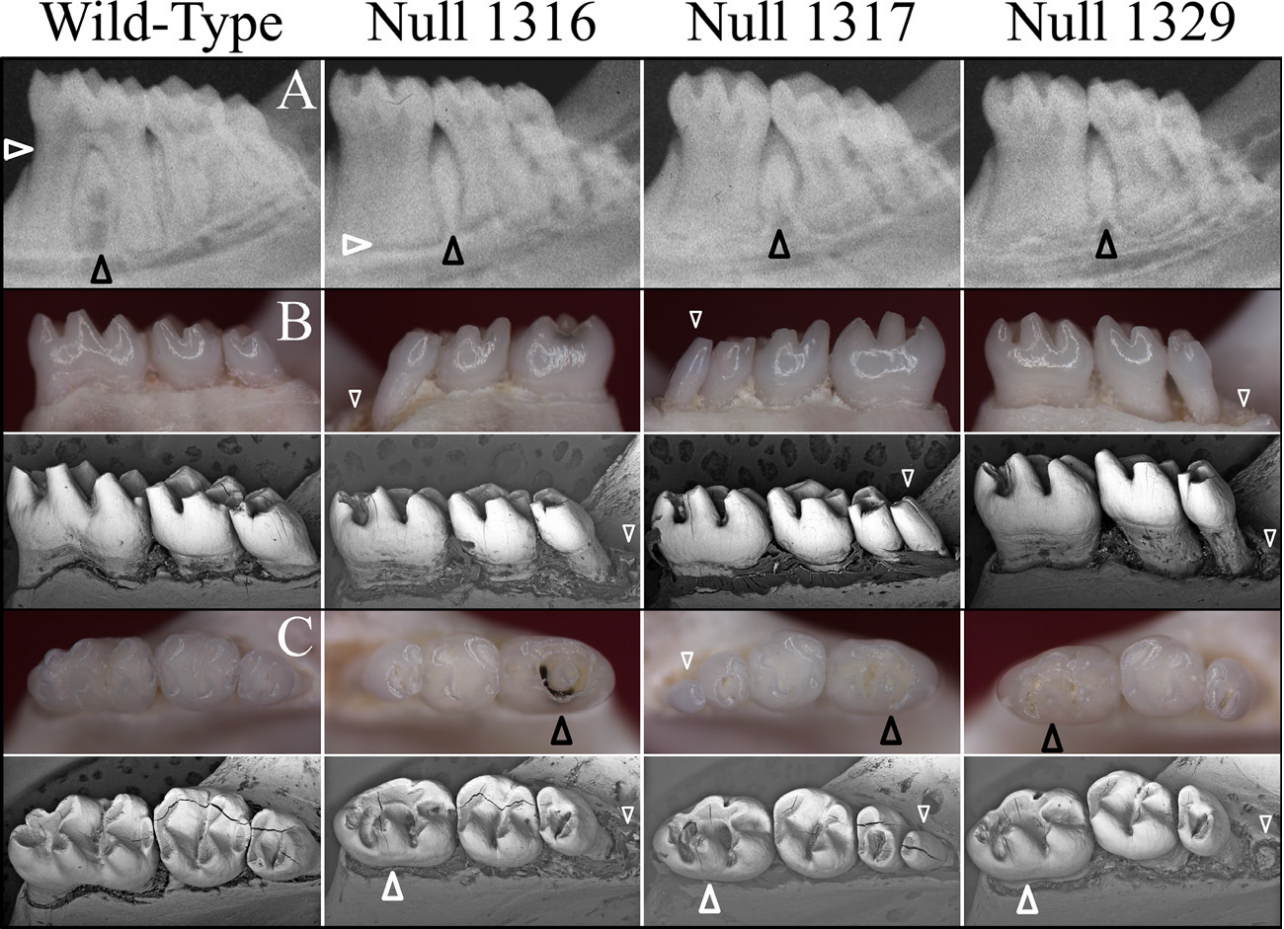
Mandibular molar morphology in *Wnt10a* null mice at 16 weeks. (A) Radiographic images of the mandibular molars show significant root dysmorphology (taurodontism) in which the bifurcation of the roots is minimal or does not occur altogether. Rightward-pointing white arrowheads mark the level of root bifuration in the mandibular first molars. Failure of the roots to separate and extend mesially or distally precluded the development of interradicular alveolar bone, but expanded the interdental alveolar bone between the first and second molars (black arrowheads). Taurodontism is also evident on the mandibular second molars, whereas the third molars are normally single-rooted. (B) Dissecting microscope (top) and scanning electron microscope (bottom) images of the lateral aspects of mandibular molars. Fourth molars or residual sockets (downward-pointing white arrowheads) where the fourth molars had been lost were observed in all of the *Wnt10a* null mice. (C) Dissecting microscope (top) and scanning electron microscope (bottom) images of the occlusal aspects of the mandibular molars. The null first molars were characterized by smaller, more rounded crowns lacking a distal cusp and by the presence of a prominent central cusp delineated by a deep groove (upward-pointing arrowheads). In the case of the 1316 null mice, the central groove was stained and apparently carious, something we have never before observed in laboratory mice. The molar occlusal surfaces of the *Wnt10a* null mice did not show any signs of attrition that would be expected if the functional properties of enamel or dentin had been reduced as a consequence of developing in the absence of WNT10A.

Wild-type mice have one incisor and three molars in each quadrant of their dentitions. Since human *WNT10A* mutations cause tooth agenesis, we expected that *Wnt10a* null mice might also exhibit missing teeth. However, the *Wnt10a* null mice have a complete dentition. More surprisingly, one mouse (Null 1317) had erupted mandibular fourth molars. The supernumerary distal molars were small in size, with a single cusp and thin root (Fig.1). Although the normal number of teeth was found in the other *Wnt10a* mice (Null 1316 and Null 1329), residual sockets for supernumerary teeth distal to mandibular third molars were evident, and indicated that fourth molars developed but were lost, presumably during tissue dissection and sample processing (Figs. S1, S2).

### Crown dysmorphologies of *Wnt10a*^*−/−*^ molars

In addition to the supernumerary mandibular molars, *Wnt10a*^*−/−*^ mice also exhibited crown dysmorphologies. To better characterize these alterations, we examined the mandibular molars of wild-type and null mice by light and scanning electron microscopy (Fig.1). The crowns of null mouse molars were smaller in size than the wild type. The mesio-distal dimension of the first molar was particularly decreased, having a “rounded-square” crown shape rather than the more “rectangular” shape of wild-type molars. The *Wnt10a*^*−/−*^ molars, especially the first molars, had a reduced area of occlusal table, not only due to the decreased mesio-distal dimensions but also because of the convergent patterns of the cusps.

Cusp patterning was altered in *Wnt10a*^*−/−*^ molars. While the mandibular first molar of wild-type mice has seven cusps (B1, B2, B3, L1, L2, L3, and 4) (Lyngstadaas et al. 1998), the most distal cusp (cusp 4) was absent from *Wnt10a* null first molars. In addition, the three buccal cusps (B1–3) and the first lingual cusp (L1) were fused together, forming a long, curved ridge surrounding the mesial and buccal aspects of the crown. The other two lingual cusps (L2 and L3) were relatively unaffected, although their size was apparently smaller. Also, instead of having a deep groove separating B2–L2 and B3–L3, the mutant first molar showed a deep U-shaped groove surrounding the second lingual cusp (L2), making this cusp the most prominent on the crown. Noticeably, this deep groove in one of the null mice (Null 1316) was black and appeared to be carious, which is exceedingly rare in mouse teeth. The overall morphology of the *Wnt10a*^*−/−*^ mandibular second molar was not significantly altered, although the tooth size appeared to be smaller. However, unlike the wild-type molars, which have five cusps (B2, B3, L2, L3, and 4), the mutant molars had only four cusps, lacking the most distal cusp (cusp 4). The *Wnt10a* null mandibular third molar had a relatively normal crown morphology and cusp patterning but appeared to be reduced in size. Taken together, these findings demonstrate that *Wnt10a* plays a significant role in early morphogenesis and patterning of tooth crown development.

### Taurodontism and root dysmorphologies of *Wnt10a*^*−/−*^ molars

**Figure 2:**
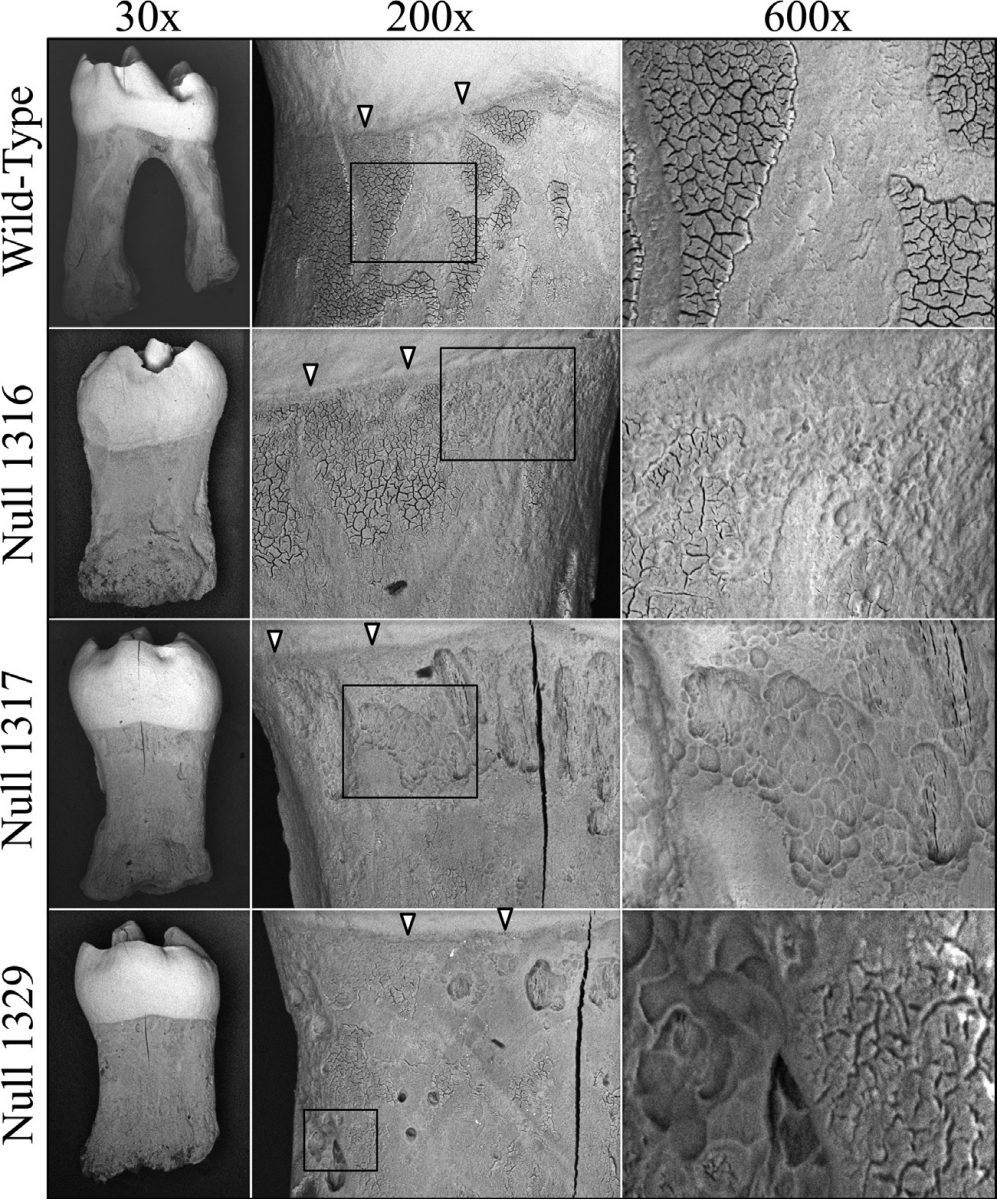
SEMs of extracted mandibular first molars at 16 weeks. Altered crown and root morphology are apparent in images taken at 30×. Higher magnitude (200×) images show the texture of the root surface below the dentino-enamel junction (DEJ, arrowheads). The 600× images magnify the boxed regions from the 200× views. Resorption lacunae of irregular in size and shape were present on the roots of the *Wnt10a* null molars, but not on the wild-type molar.

Besides altered crown morphology, *Wnt10a*^*−/−*^ molars showed taurodontic root morphology (Figs.2). Unlike the wild-type molars, which have a short root trunk and a high furcation dividing the mesial and distal roots, the *Wnt10a*^*−/−*^ molars had elongated root trunks with a low, or absent, furcation. This characteristic was observed in the mandibular first and second molars of all three null mice. Correspondingly, the pulp chambers of these null molars were vertically elongated with very little root canals, resulting in a torch-shaped morphology. Furthermore, the scanning electron micrographs of the extracted molars revealed many irregular concavities, resembling osteoclast lacunae on the root surfaces of *Wnt10a*^*−/−*^ molars, which left a rougher root surface compared to the wild-type molars (Fig.2). External root resorption was observed in all three null mice. It appeared in patches in all areas of the root surface, was especially apparent in the Null 1317 and Null 1329 mice, and was not observed in WT mice.

**Figure 3:**
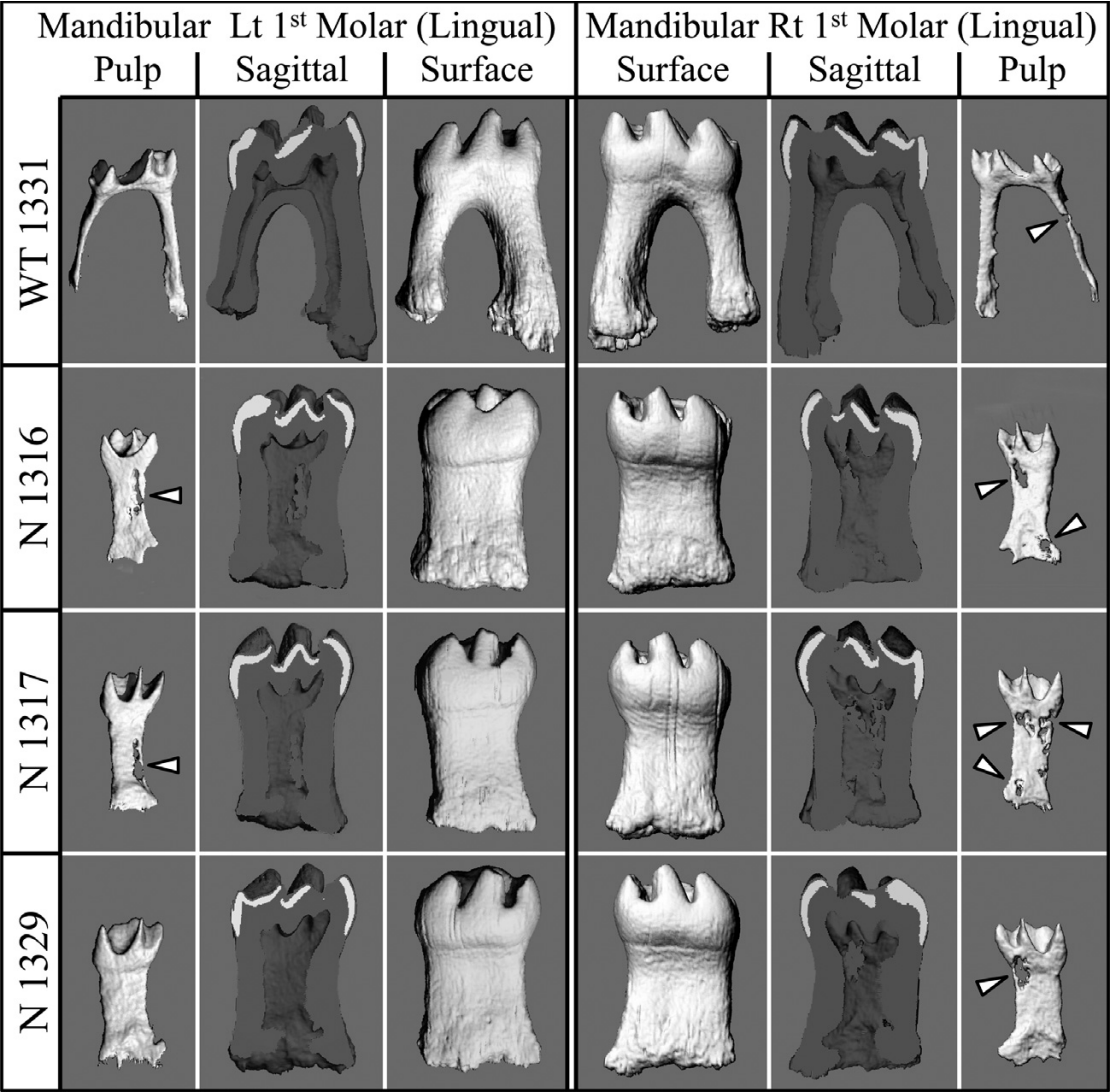
MicroCT images of the right and left mandibular first molars from 16-week-old wild-type and *Wnt10a* null mice. The 3D *μ*CT images were generated for the mandibular first molars scanned in situ (within the hemimandibles). The teeth were outlined to focus on the dental structures. Hard tissue and soft tissues were imaged by setting the threshold above or below 260, respectively. The sagittal views revealed irregular mineral structures that corresponded to vacancies within pulpal tissue (arrowheads). These are pulp calcifications or “pulp stones”, which are more extensive in the molars of the *Wnt10a* null mice than in the wild type. Raising the threshold above 260 did not selectively remove the pulp stones, indicating that they are approximately the same density as dentin.

We also performed X-ray *μ*CT to better characterize the morphology and structures of the molars. Consistent with what we observed using traditional radiography and SEM, the 3D reconstructions showed taurodontism of the *Wnt10a*^*−/−*^ mandibular first molar roots (Fig.3). Sagittal sections and 3D reconstructions of pulp morphology revealed that the mutant molars had elongated torch-shaped pulp chambers, in which some pulp calcifications (pulp stones) were observed. These findings suggest that *Wnt10a* plays an important role in root development, especially furcation formation, and that a loss of *Wnt10a* function leads to a taurodontic root morphology.

### *μ*CT density and volume measurements of *Wnt10a*^*−/−*^ first molars

**Figure 4:**
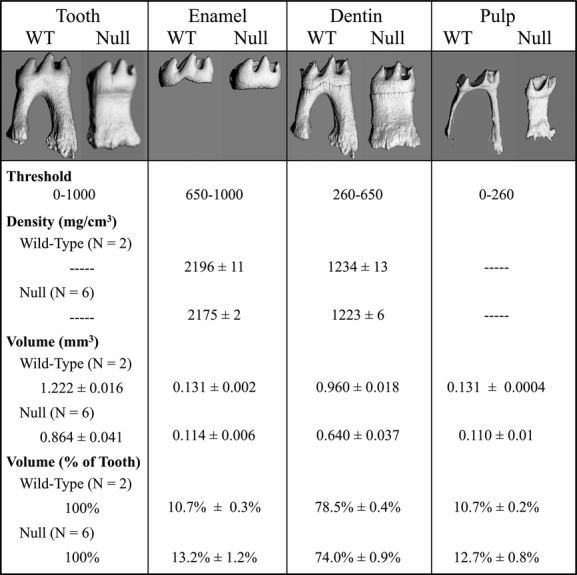
MicroCT analyses of mineral density and volume of mandibular first molars at 16 weeks. The whole tooth, enamel, dentin, and pulp were individually isolated by varying the density threshold for detection. The density measurements were similar in the *Wnt10a* null and wild-type mice, with the density of enamel and dentin in the null molars being over 99% as dense as the enamel and dentin of the wild type. The null first molars were smaller than the wild type: having only 71% as much volume. The reduction in the volume of dentin was slightly greater than the reductions in the volumes of enamel and pulp, so that the enamel and pulp comprised about 2.5% and 2% more of the volume of null molars compared to that of the wild type, respectively.

To further characterize the structural alteration of *Wnt10a*^*−/−*^ first molars, we measured the average densities of the enamel and dentin, and the volumes of the whole tooth, enamel, dentin, and pulp of mandibular first molars. Individual tissues were isolated by setting specific ranges of *μ*CT threshold values: 0–260 for pulp, 260–650 for dentin, 650–1000 for enamel, and 0–1000 for the whole tooth (Fig.4). The means of the measurements were calculated for the two wild-type and the six null mandibular first molars. The results show that the null molar densities of enamel and dentin are comparable to those of the wild-type molars, with both the enamel and dentin showing density reductions of less than 1%.

The total tooth volume (size) of the *Wnt10a*^*−/−*^ first mandibular molars average 71% of the wild-type, demonstrating a microdontic phenotype of the *Wnt10a* null teeth, which is consistent with our SEM observations. The null enamel, dentin, and pulp volumes averaged 87%, 67%, and 84% of those of the wild-type molars, respectively, so diminished dentin volume is the predominant factor in the reduction of total tooth volume. The disproportionate loss of dentin volume altered the proportions of the enamel, dentin, and pulp components in the null relative to the wild-type molars. The enamel volume as a percentage of the total tooth volume increased from 10.7% to 13.2%, and the pulp increased from 10.7% to 12.7% in the null molars.

Taken together, these studies indicate that the absence of *Wnt10a* during mouse molar development results in supernumerary mandibular fourth molars, smaller molars, altered root form (taurodontism), reduced volume of dentin, pulp calcifications, molar crown dysmorphologies, and increased risk of root resorption following tooth development and eruption.

### *Wnt10a*^*−/−*^ incisors

**Figure 5:**
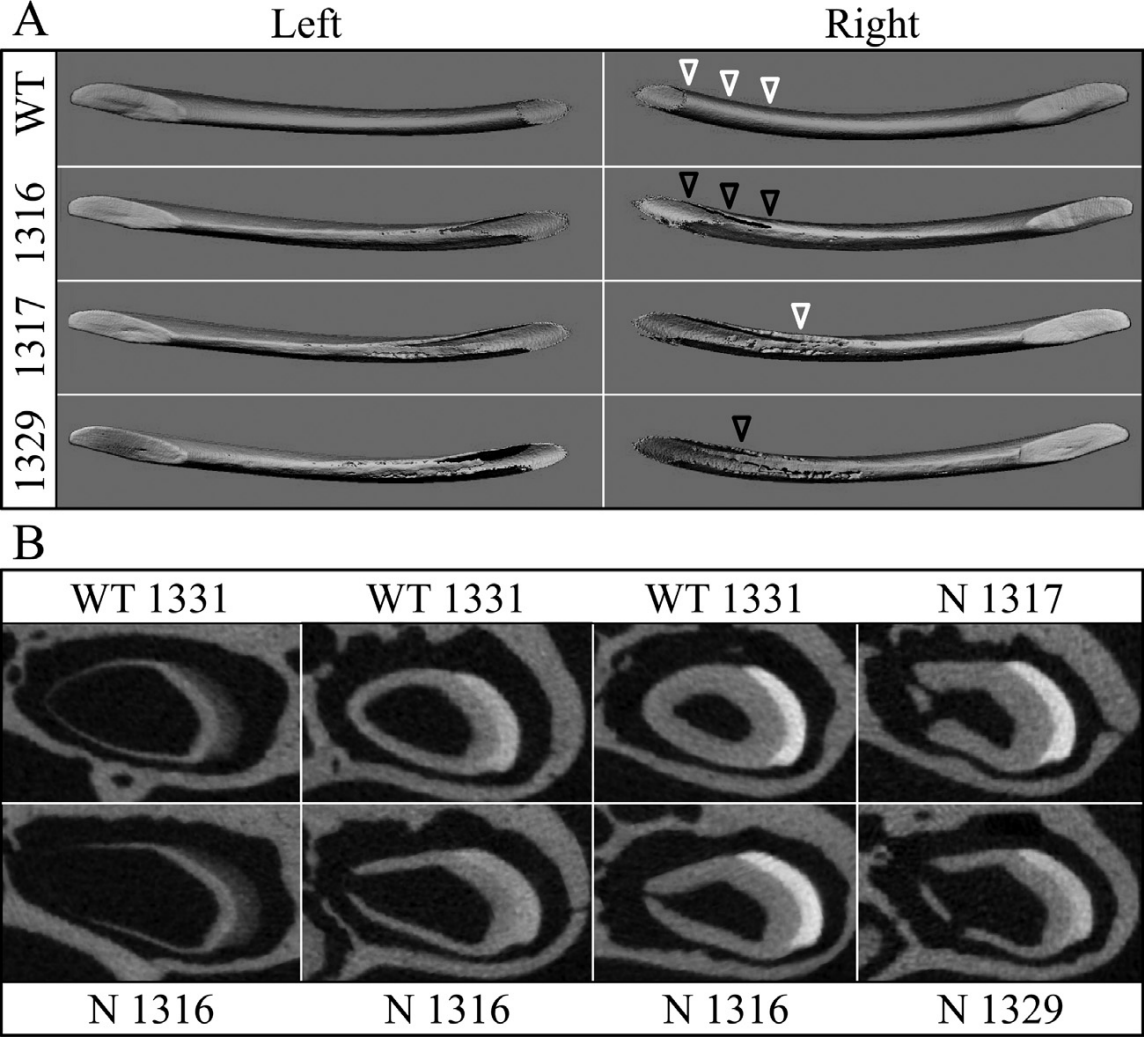
MicroCT analysis of mandibular incisors at 16 weeks. (A) 3D reconstructions of mandibular incisors at 16 weeks. Orientations: The apical ends of the incisors are toward the center; incisal tips are toward the periphery; lingual (dentin) side is up (toward the viewer); buccal (enamel) side is down and not visible. All incisors from the *Wnt10a* null mice showed a failure to close in their apical–lingual region. In Null 1316 a single wedge-shaped defect extended from the apical end and narrowed incisally. In the Null 1317 and Null 1329 mice, the defects extended further toward the middle of the incisors, with a thin strip of dentin running at the center of the defects. Thus, in the Null 1316, there is a single wedge-shaped deficiency, whereas in Null 1317 and Null 1329, there are two overlapping wedge-shaped deficiencies. White arrowheads and black arrowheads mark the positions of 2D cross sections shown in the top and bottom rows of part B, respectively. (B) Cross sections from apical ends of the incisors. The wild-type incisor has a closed circle of dentin at its apical end. The *Wnt10a* Null 1316 incisor cross sections shows a single gap on the lingual aspect of the mandibular incisor that narrows from apical to incisal. The Null 1317 and 1329 incisor cross sections show two openings on the lingual face corresponding to the two wedge-shaped deficiencies in the lingual dentin.

*Wnt10a*^*−/−*^ incisors were characterized using *μ*CT. In wild-type incisors, dentin completely encloses the pulp lingually (opposite the enamel). 3D reconstructions of the mandibular incisors showed that the lengths of the WT and *Wnt10a* null incisors were similar but, significantly, the lingual side of the apical end of the *Wnt10a*^*−/−*^ incisor had a V-shaped density defect, suggesting that the onset of dentin mineralization at the lingual surface of mandibular incisor was delayed or defective (Fig.5A). This was also evident in the *μ*CT cross sections from the apical ends of the incisors (Fig.5B). The cross sections from various levels in the discontinuous region showed two alternative patterns: with either a single gap lingually, or two gaps, depending upon whether a single or double wedge deficiency had formed in the absence of WNT10A.

### *WNT10A* mutations and isolated tooth agenesis

**Figure 6:**
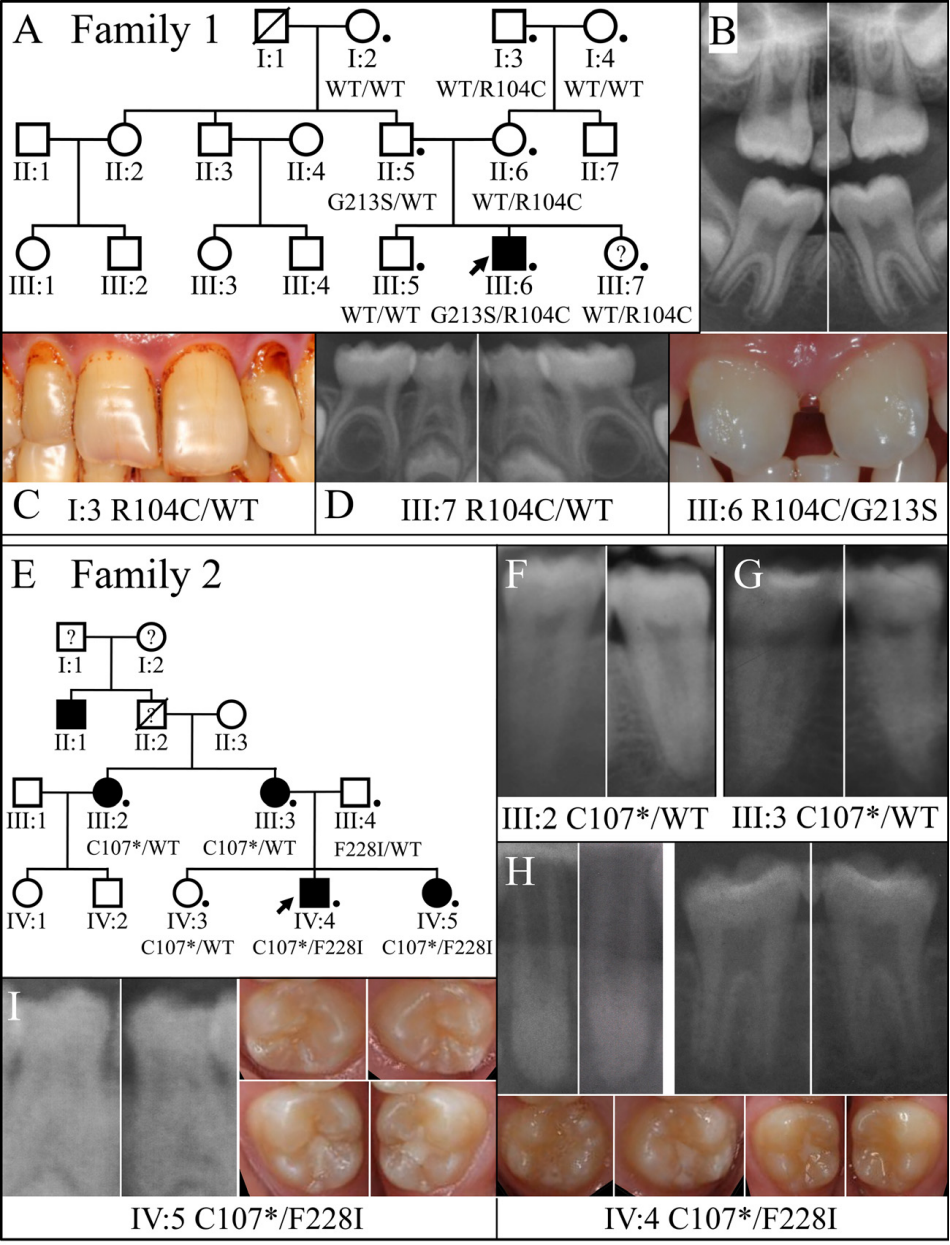
Pedigrees and anomalous dental morphologies in persons with *WNT10A* defects: Families 1 and 2. (A) Pedigree of Family 1. A dot marks each of the eight individuals that were recruited and evaluated. The *WNT10A* genotype is shown for each recruited member. Only the proband (III:6) had developmentally absent teeth (18 permanent teeth) and was the only individual with defects in both *WNT10A* alleles (p.Arg104Cys/p.Gly213Ser). (B) Detail from the proband's (III:6) panorex radiograph showing taurodontism in all first molars (#3, 14, 19, 30) (top). Oral photograph showing the proband's misshapened central incisors (bottom). (C) Oral photograph of proband's maternal grandfather (I:3), who was heterozygous for the p.R104C defect in *WNT10A*, showing peg lateral incisors (#7, 10). (D) Details from the panorex radiograph of the proband's younger sister (III:7), who was heterozygous for the p.Arg104Cys defect in *WNT10A*, showing taurodontism in all primary mandibular first molars (#K, L, S, T). (E) Pedigree of Family 2. A dot marks each of the six individuals that were recruited and evaluated. The *WNT10A* genotype is shown for each recruited member. Affected members had developmentally missing teeth: III:2 (2 teeth); III:3 (1 tooth); IV:4 (8 teeth); IV:5 (13 teeth). (F) Details from the panorex radiograph of the proband's maternal aunt (III:2), who was heterozygous for the p.Cys107*WNT10A defect, showing taurodontism in the mandibular second molars (#17, 31). (G) Details from the panorex radiograph of the proband's mother (III:3), who was heterozygous for the p.Cys107*WNT10A defect, showing taurodontism in the mandibular second molars (#18, 31). (H) Details from the proband's (IV:4) panorex radiograph, who had defects in both *WNT10A* alleles (p.Cys104*/p.Phe228Ile), showing peg lateral incisors (left; #7, 10) and taurodontism in the mandibular first molars (right; #19, 30). Oral photographs show rounded first molar morphology, with maxillary first molars (left; #3, 15) lacking the distal palatal cusp and the mandibular first molars (right; #19, 30) lacking the distal cusp. (I) Details from the proband's younger sister's (IV:5) panorex radiograph, who also had defects in both *WNT10A* alleles (p.Cys104*/p.Phe228Ile), showing taurodontism in the primary mandibular first molars (right; #L, S). Oral photographs show rounded first molar morphology, with maxillary first molars (left; #3, 15) lacking the distal palatal cusp and the mandibular first molars (right; 19, 30) lacking the distal cusp.

**Figure 7:**
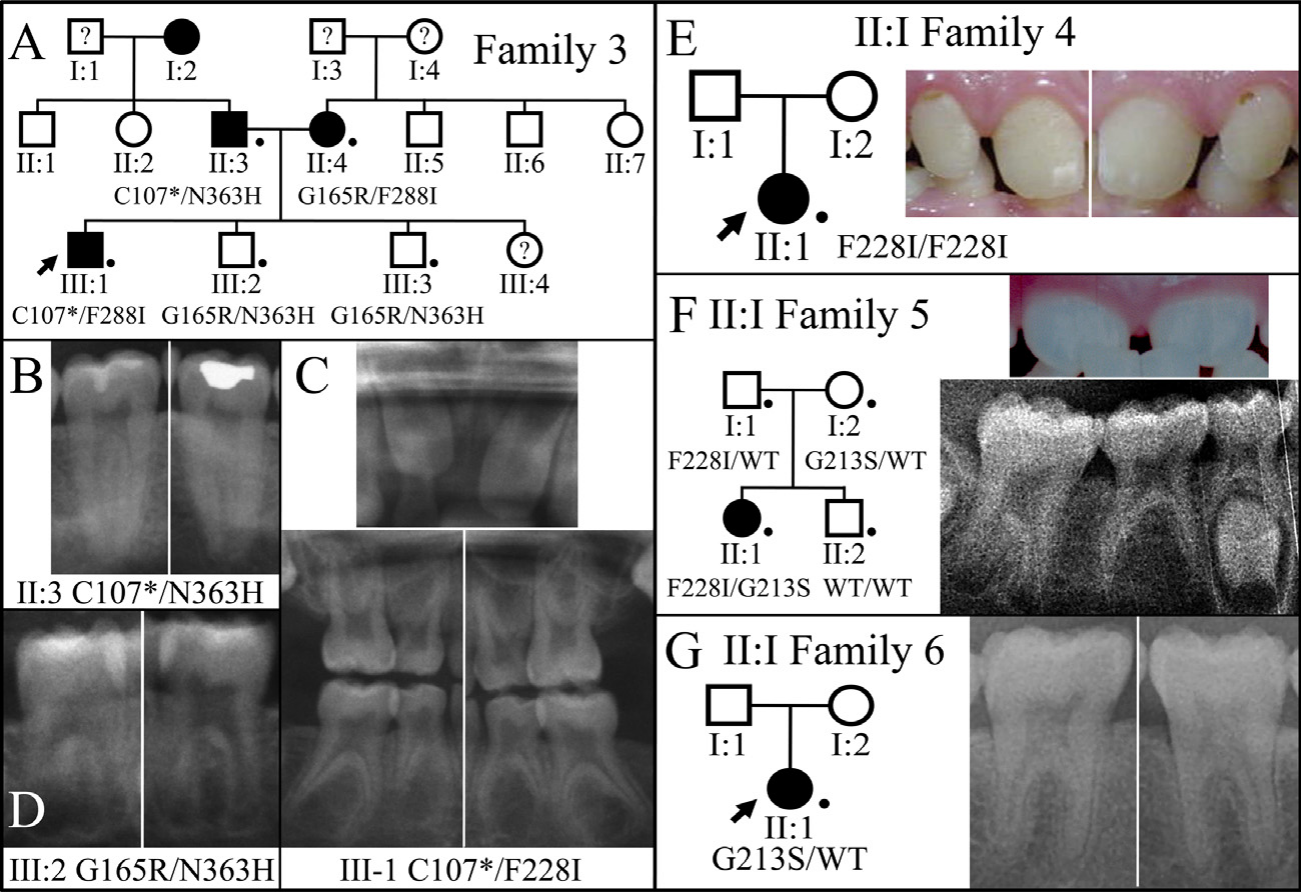
Pedigrees and anomalous dental morphologies in persons with *WNT10A* defects: Families 3 through 6. (A) Pedigree of Family 3. A dot marks each of the five individuals that were recruited and evaluated. The *WNT10A* genotype is shown for each recruited member. All four *WNT10A* alleles in the proband's parents carried different *WNT10A* sequence variations. Affected members with developmentally missing teeth were: II:3 (6 teeth); II:4 (2 teeth); III:1 (23 teeth). (B) Details from the panorex radiograph of the proband's father (II:3) showing taurodontism in the mandibular second molars (#18, 31). (C) Details from the proband's (III:2) panorex radiograph showing conical maxillary central incisors (top; #8, 9) taurodontism in all primary first and second molars (#A, B, I, J, K, L, S, T). (D) Details from the panorex radiograph of the proband's younger brother (III:2) showing taurodontism in the mandibular first molars (#19, 30). (E) Pedigree of Family 4 and oral photograph of the proband (II:1) who had 17 developmentally absent teeth and peg-shaped maxillary incisors (#7, 8, 9, 10). (F) Pedigree of Family 5 and detail from the proband's (II:1) panorex radiograph showing taurodontism of the mandibular second molars (#18, 31). The proband had 11 developmentally absent teeth and peg-shaped maxillary central incisors (#8, 9). (G) Pedigree of Family 6 and detail from the proband's (II:1) panorex radiograph showing taurodontism of the mandibular first molars (#19, 30). The proband had three developmentally absent teeth.

We characterized six families with isolated (nonsyndromic) tooth agenesis associated with *WNT10A* defects. Combined, there were 11 individuals with tooth agenesis ranging from 1 to 23 teeth (excluding third molars) that failed to develop (Table1). In addition to the absence of teeth, persons with *WNT10A* defects showed dental crown and root dysmorphologies including peg lateral and/or central incisors, altered molar cusp patterns and crown morphology, and root taurodontism (Figs.6, 7).

**Table 1:** Dental finding in six families with *WNT10A* mutuations.

	8	7	6	5	4	3	2	1	1	2	3	4	5	6	7	8	*WNT10A*	Notes
Family 1																		
I:3	?															?	p.R104C	1
I:3	?															?	WT	
II:5																	WT	
II:5																	p.G213S	
II:6	?															?	p.R104C	
II:6	?															?	WT	
III:6	?			*	*	*	*			*	*	*	*			?	p.R104C	2
III:6	?			*	*	*	*	*	*	*	*	*	*			?	p.G213S	
III:7	?			?									?			?	p.R104C	3
III:7	?															?	WT	
Family 2																		
III:2	*															*	p.C107*	
III:2	*			*									*			*	WT	4
III:3	*			*												*	p.C107*	
III:3	*															*	WT	5
III:4																	WT	
III:4																	p.F228I	
IV:3	*															*	p.C107*	
IV:3																	WT	
IV:4	?	*		*									*		*	?	p.C107*	6
IV:4	?	*						*	*						*	?	p.F228I	6
IV:5	?			*			*			*		*	*		*	?	p.C107*	7
IV:5	?			*			*	*	*	*			*		*	?	p.F228I	7
Family 3																		
II:3					*		*			*		*					p.C107*	8
II:3								*	*								p.N363H	8
II:4	*	*															p.G165R	
II:4	*	*															p.F228I	
III:1	?		*	*	*	*	*			*	*	*	*	*		?	p.C107*	9
III:1	?		*	*	*	*	*	*	*	*	*	*	*	*	*	?	p.F228I	9
III:2	?															?	p.G165R	10
III:2	?															?	p.N363H	10
III:3	?															?	p.G165R	
III:3	?															?	p.N363H	
Family 4–6																		
II:1	?	*		*	*	*					*	*	*		*	?	p.F228I	11
II:1	?	*		*	*	*	*			*	*	*	*			?	p.F228I	
II:1	?		*	*	*							*	*	*		?	p.F228I	12
II:1	?			*				*	*				*	*		?	p.G213S	12
II:1	*					*					*					*	p.G213S	
II:1	*			*												*	WT	14

* Tooth never formed; E, tooth was extracted; ?, unknown if tooth will form because of age at the time of the radiograph.

1 I:3 Peg laterals (7 and 10) Curly hair, but also in III:5, which is WT.

2 III:6 has “microdontia” of 8 and 9. All first molars have taurodontism (3, 14, 19, 30).

3 III:7 has taurodontism (primary mandibular first and second molars).

4 III:2 Mandibular second Molars have taurodontism.

5 III:3 Mandibular second Molars have taurodontism.

6 IV:4 Peg lateral (7 and 10); 19 and 30 taurodontism. Photos: all first molars (Max: distal palatal; Mand: distal cusps are missing)

7 IV:5 8, 9 (Microdontia); L and S taurodontism. Photos: all first molars (Max: distal palatal; Mand: distal cusps are missing)

8 II:3 18 and 31 have taurodontism.

9 III:1 All first and second primary molars have taurodontism. Conical maxillary central incisors.

10 III:2 Taurodontism on 19 and 30.

11 Peg-shaped maxillary incisors.

12 II:1 18 and 31 have taurodontism.

13 Heterozygous parents of proband reported no missing teeth (unconfirmed).

14 II:1 Taurodontism of the mandibular first and second molars.

### Family 1

Family 1 is a three-generation Taiwanese family (Fig.6A). The proband (III:6) was an 8.5-year-old boy who had 18 permanent teeth missing, excluding third molars (tooth numbers 4, 5, 6, 7, 10, 11, 12, 13, 20, 21, 22, 23, 24, 25, 26, 27, 28, 29). Clinically, his maxillary central incisors showed abnormal crown morphology with the mesiodistal dimension tapering from the cervical to the incisal (Fig.6B). The crown morphology of the permanent first molars was within normal limits. However, the panoramic radiograph revealed that all of these four permanent first molars were taurodontic, with elongated pulp chambers (Fig.6B). The proband was the only affected individual in the family. Other family members had no missing teeth excepting third molars (Figs. S3–S8), although multiple teeth were reportedly extracted in the individuals of the first generation (I:2, I:3, I:4). However, the maternal grandfather (I:3) had peg lateral incisors, and the youngest child (III:7) had taurodontic mandibular primary molars, although they did not show a tooth agenesis phenotype.

No hair, skin, or sweating problems were observed in the family, except that the paternal grandmother (I:2) was reported to have thin hair, and the scalp hair of the probands parents (II:5, II:6) was curly. Noticeably, among the five recruited individuals of the first two generations, the maternal grandfather (I:3) and the father (II:5) had intestinal polyps, while the other three (I:2, I:4, II:6) did not, according to the results of colonoscopy.

Based upon the family pedigree, we suspected that the tooth agenesis might be caused by a recessive mutation. Therefore, we performed whole-exome sequencing of the parent–child trio (II:5, II:6, III:6). By screening through the candidate genes of tooth agenesis with exome data, we identified no potential disease-causing sequence variants in *MSX1*,*PAX9*,*AXIN2*,*LTBP3*,*EDA*,*EDAR*, and *EDARADD*, but did identify two missense mutations in *WNT10A* (g.6825C>T, c.310C>T, p.Arg104Cys; g.14712T>A, c.637T>A, p.Gly213Ser). The p.Arg104Cys variation is not listed in the Exome Variant Server (EVS) database (DB). The p.Gly213Ser variation shows almost zero frequency (1/13,006) in the EVS DB. Sanger sequencing demonstrated that the proband was a compound heterozygote for these two mutations with the p.Arg104Cys mutation inherited from the mother and the p.Gly213Ser from the father. Noticeably, the maternal grandfather (I:3) and the youngest child (III:7), who had peg laterals and molar taurodontism. respectively, carried the heterozygous p.Arg104Cys mutation. While the p.Gly213Ser mutation has been shown to be disease causing (Table S1), the p.Arg104Cys mutation is a novel mutation that has not been previously reported. This missense mutation substituted a highly conserved positively charged residue (Arg^104^) with cysteine, which was predicted to be probably damaging with a score of 1.000 by PolyPhen-2. Therefore, we conclude that the p.Arg104Cys mutation is disease causing. Although some of the heterozygous carriers of this mutation (I:3, III:7) displayed abnormal tooth morphology, none of them had missing teeth except third molars, which suggested a low disease penetrance and expressivity for tooth agenesis associated with this *WNT10A* allele.

### Family 2

Family 2 was a four-generation Caucasian family referred to us by a geneticist (Fig.6E–I). Based upon the clinical impression, the geneticist ruled out ectodermal dysplasia, and isolated tooth agenesis was diagnosed for the family. The proband (IV:4) was a 10-year-old boy who had eight missing teeth (tooth numbers 2, 4, 13, 15, 18, 24, 25, 31) excluding third molars (Fig. S9). His younger sister (IV:5) was also affected with a total of 15 missing teeth (tooth numbers 2, 4, 7, 10, 12, 13, 15, 18, 20, 23, 24, 25, 26, 29, 31), whereas the older sister (IV:3) had all permanent teeth except two missing upper third molars. The disease trait seemed to come from the maternal side, since the mother (III:3) and the aunt (III:2) were missing one (tooth number 4) and two (tooth numbers 20, 29) teeth, respectively, whereas the father (III:4) was not affected and had full set of permanent teeth. These findings suggested a dominant pattern of disease inheritance and a variation in disease expressivity (severity) between generations (Figs. S10–S12).

In addition to tooth number abnormality, altered crown and root morphology of teeth were also observed in different family members. For the proband (IV:4) (Fig.6H), the upper lateral incisors were microdontic (peg laterals). His mandibular first molars were missing the distal cusps, while the maxillary ones had prominent mesio-palatal cusps but no disto-palatal cusps, which made the teeth heart shaped. Radiographically, the lower first molars exhibited taurodontism. For the younger sister (IV:5) (Fig.6I), her upper central incisors were microdontic, and the first molars had similar characteristics to those of the proband's teeth. Also, her mandibular primary first molars appeared taurodontic. For the mother and the aunt, all their mandibular second molars showed taurodontism on panoramic radiographs. Besides these dental phenotypes, no other extra-dental abnormalities were noted, except that the proband's mother reported slow hair growth in the proband, and the probands' father and younger sister.

To identify the genetic defect causing tooth agenesis in this family, we submitted DNA samples from four of the family members (III:2, III:4, IV:4, IV:5) for exome sequencing. With the analyzed exome data, we first searched for mutations in genes known to be associated with tooth agenesis and found two reported *WNT10A* mutations, g.6836C>A, c.321C>A, p.Cys107* and g.14757T>A, c.682T>A, p.Phe228Ile. The p.Cys107* variation shows ∼0.10% frequency (13/13,006), and the p.Phe228Ile variation ∼1.8% frequency (241/13,006) in the EVS DB. No potential disease-causing mutations were identified in other candidate genes for isolated tooth agenesis, *MSX1*,*PAX9*,*AXIN2*,*LTBP3*,*EDA*,*EDAR*, and *EDARADD*. The *WNT10A* mutations were confirmed and segregation analyzed by Sanger sequencing. The results showed that while the mother (III:3) and the aunt (III:2) were heterozygous for the nonsense mutation (p.Cys107*), both of the two affected children (IV:4, IV:5) were compound heterozygotes for p.Cys107* and p.Phe228Ile. Interestingly, the unaffected older sister (IV:3) and the father (III:4) were heterozygous carriers of the p.Cys107* and p.Phe228Ile mutation, respectively, which suggested incomplete penetrance of tooth agenesis for both of these *WNT10A* mutations in heterozygotes.

### Family 3

Family 3 was a three-generation Caucasian family referred to us by a geneticist (Fig.7). The proband (III:1) at age 6 was found to have 23 missing permanent teeth (excluding third molars). Only permanent tooth numbers 2, 8, 9, 15, and 31 could be detected radiographically (Fig. S13). His primary teeth were greatly spaced, suggesting a microdontia phenotype (Fig. S13). Also, taurodontism was apparent in all primary molars (Fig.7C). The proband's parents were also affected. While the father (II:3) had six missing teeth (tooth numbers 5, 7, 10, 12, 24, 25), the mother (II:4) was missing right upper and lower second molars as well as all the third molars. Two of proband's siblings (III:2 and III:3) were not affected, and the youngest child was too young to determine the disease status. The panoramic radiographs revealed that the lower second molars of the father (II:3) and the first permanent molars of the second child (III:2) were taurodontic. Ectodermal dysplasia was ruled out by the geneticist based upon a thorough physical examination, although the mother reported that her children had excessive sweating over their hands and feet. Other medical and dental histories of the family reported by the parents were not contributory.

Based upon the pattern of disease inheritance as well as the phenotypic similarity of this family with the previous two families, we specifically targeted *WNT10A* by Sanger sequencing. However, considering the potential phenotypic contribution from sequence variants of other ectodermal dysplasia genes, we also analyzed *EDA*,*EDAR*, and *EDARADD*. The results identified four *WNT10A* sequence variations running in this family, g.6836C>A, c.321C>A, p.Cys107*; g.7008G>A, c.493G>A, p.Gly165Arg; g.14757T>A, c.682T>A, p.Phe228Ile; and g.15162A>C, c.1087A>C, p.Asn363His (Fig. S14). The p.Gly165Arg variation shows ∼0.78% frequency (102/13006) and the p.Asn363His variation shows ∼0.028% frequency (3/10642) in the EVS DB. Since both parents were compound heterozygous for two *WNT10A* mutations, all of the children were also compound heterozygous for two of the four *WNT10A* alleles. The father (II:3) and the mother (II:4) carried p.Cys107*, p.Asn363His, and p.Gly165Arg, p.Phe228Ile mutations, respectively. The proband (III:1) was a compound heterozygote for p.Cys107* and p.Phe228Ile mutations, while the two unaffected children (III:2, III:3) were both carrying p.Gly165Arg and p.Asn363His mutations.

### Family 4

Family 4 was Caucasian, with an apparent simplex pattern of inheritance (Fig.7E), as both parents denied having any missing teeth. The proband was missing 17 teeth (tooth numbers 2, 4, 5, 6, 11, 12, 13, 15, 20, 21, 22, 23 26, 27, 28, 29, 31) excluding third molars. Target gene analyses of *WNT10A*,*EDA*,*EDAR*, and *EDARADD* revealed that the proband was homozygous for the *WNT10A* p.Phe228Ile mutation (Fig. S15). Besides the missing teeth, the proband had peg-shaped maxillary incisors (tooth numbers 7, 8, 9, 10). Abnormalities of hair, nails, or sweating were denied.

### Family 5

Family 5 was a Hispanic nuclear family with parents and two children. The proband, an 8-year-old girl, was the only affected individual in the family, who had 11 missing teeth (tooth numbers 3, 4, 5, 12, 13, 14, 19, 20, 24, 25, 29) excluding third molars. All of her primary molars and the only permanent first molar were taurodontic (Fig.7F). Also, her maxillary central incisors showed abnormal crown morphology, with the mesiodistal dimension tapering from the cervical to the incisal. The mother reported that the proband is otherwise healthy. The family medical and dental histories were not contributory. Abnormalities of hair, nails, or sweating were denied.

Target gene analyses of *WNT10A*,*EDA*,*EDAR*, and *EDARADD* revealed that the proband was compound heterozygous for two *WNT10A* missense mutations, g.14712T>A, c.637T>A, p.Gly213Ser and g.14757T>A, c.682T>A, p.Phe228Ile (Fig. S16). The p.Gly213Ser mutation was inherited from the mother, and the p.Phe228Ile from the father. The younger brother had neither *WNT10A* mutation (Fig. S17).

### Family 6

The proband of Family 6, a 35-year-old lady, was a sporadic case from Taiwan. Her maxillary canines, right mandibular second bicuspid, and all third molars were missing. The upper left lateral incisor was microdontic, and taurodontism was evident on all molars. Clinically, no additional morphological alterations of teeth were observed. She reported no apparent hair, skin, nail, or sweating problems. With *WNT10A*,*EDA*,*EDAR*, and *EDARADD* being screened, a heterozygous p.Gly213Ser mutation was identified (Fig. S18). Although the p.Gly213Ser variation has a very low frequency (1/13,006) in the EVS DB, it has a higher allele frequency in Asian population, where it was associated with the agenesis of maxillary permanent canines (Kantaputra et al. 2014).

## Discussion

Wnt/*β*-catenin signaling is critical for organogenesis of many tissues, including teeth (Liu and Millar 2010; Lan et al. 2014). The finding that human *WNT10A* mutations in particular cause tooth agenesis not only reaffirms the importance of Wnt/*β*-catenin signaling but also indicates critical roles for specific Wnt molecules during tooth development. The reported disease-causing *WNT10A* mutations include nonsense, missense, and frameshift mutations distributed over all of the four exons (Table S1), which suggests a pathogenic mechanism of loss of function and haploinsufficiency. Conditional deletion of *β*-catenin in either the dental epithelium or mesenchyme in mice leads to tooth developmental arrest at the bud stage. This helps explain the presence of tooth agenesis in *WNT10A* patients (Liu et al. 2008; Chen et al. 2009). However, inconsistent with the human phenotypes, *Wnt10a* null mice exhibit a complete dentition with an extra tooth distal to the third mandibular molars (4th molars). Similarly, while mutations in *axis inhibitor 2* (*AXIN2*; OMIM *604025) and latent transforming growth factor-beta-binding protein 3 (*LTBP3*; OMIM *602090) were demonstrated to cause severe oligodontia in humans (Lammi et al. 2004; Noor et al. 2009), neither *Axin2* nor *Ltbp3* null mice show tooth agenesis (Dabovic et al. 2002; Yu et al. 2005). This phenotypic discrepancy raises the concern that mice might not be the most appropriate animal model to study human tooth agenesis. While humans have two dentitions (primary and secondary), rodents have only one, which is more analogous to the primary dentition in humans. Also, compared with humans, rodents have a reduced dentition with only one incisor and three molars per quadrant. As human tooth agenesis usually affects the secondary (permanent) dentition rather than the primary dentition (Nieminen 2009) the molecular mechanisms for formation of two dentitions are not identical, so genetically engineered mice might only partially phenocopy human tooth agenesis and provide an uncertain model for investigating its disease mechanisms. Humans have the dental formulae of I_2_-C_1-_M_2_ and I_2_-C_1_-P_2_-M_3_ in primary and secondary dentitions, respectively. While primary teeth initiate from de novo dental laminae, the succeeding permanent teeth develop from the dental laminae of the preceding primary teeth (Jernvall and Thesleff 2012). Also, teeth from the same class are thought to derive from the same original dental lamina, which extends distally and gives rise to teeth with similar tooth morphology. Although it was claimed that there is no specific pattern of missing teeth in *WNT10A*-associated tooth agenesis (van den Boogaard et al. 2012; Plaisancie et al. 2013), some reported that *WNT10A* mutations cause tooth agenesis with lateral incisors and second premolars being frequently involved (Kantaputra and Sripathomsawat 2011; Song et al. 2014), suggesting that *WNT10A* might be important for extension of dental lamina and the process of serial addition of teeth rather than de novo tooth formation.

Despite the known differences between humans and mice with regard to their dentitions, the finding that *Wnt10a* null mice have supernumerary teeth is puzzling. It has been shown that inactivation of Wnt/*β*-catenin signaling in mice abolishes tooth development at an early stage, whereas overactivation of Wnt/*β*-catenin signaling leads to the formation of multiple ectopic teeth (Sasaki et al. 2005; Liu et al. 2008). However, these results only demonstrate the significance of the canonical Wnt signaling pathway (Wnt/*β*-catenin signaling) in tooth formation. Therefore, it is possible that WNT10A might be involved in noncanonical Wnt signaling, which might have a distinct role during odontogenesis. Many mouse models have supernumerary teeth, including knockout mice for genes involved in BMP, FGF, and SHH signaling, demonstrating that tooth number is determined by a complicated genetic network of many signaling pathways that are critical for early tooth development (Wang and Fan 2011). Noticeably, while most of these mice exhibit supernumerary teeth located at the diastema region mesial to the first molars, the extra teeth of *Wnt10a* null mice are at the end of the dentition, distal to the third molars, which suggests that the underlying mechanism of supernumerary tooth formation in *Wnt10a* null mice might be distinct from those of other mouse models. Based upon the model of serial addition of mammalian molars, loss of *Wnt10a* function somehow extends the odontogenic potential of dental laminae of the third molars and leads to formation of distal molars. Alternatively, it is possible that loss of *Wnt10a* function splits the third molar tooth germ to give rise to two separate teeth, since the last two molars of *Wnt10a* null mice appear to be abnormally small. Further investigations need to be conducted to unravel the underlying mechanism of distal molar formation in *Wnt10a* null mice.

The mandibular first and second molars of *Wnt10a*^*−/−*^ mice were missing the most distal cusps, and appeared to phenocopy the altered molar crown morphology we observed in Family 2. Also, the microdontic phenotype in *Wnt10a*^*−/−*^ mice was consistent with the high prevalence of peg laterals and misshapened central incisors in *WNT10A* patients. The finding that the teeth of *WNT10A* patients and *Wnt10a* null mice both show abnormalities in tooth size and cusp morphology demonstrates a significant role of WNT10A in tooth morphogenesis. The primary enamel knot, an epithelial structure of the enamel organ that functions as a signaling center during early tooth development, regulates the growth and folding of the dental epithelium and influences formation of the secondary enamel knot that patterns molar crown development (Lan et al. 2014). The specific expression of *Wnt10a* in primary and secondary enamel knots during murine tooth formation explains the aberrant cusp patterning when *WNT10A* is mutated (Dassule and McMahon 1998). Conditional inactivation of Wnt/*β*-catenin signaling following the bud stage of murine odontogenesis causes formation of blunted molar cusps (Liu et al. 2008), suggesting that WNT10A regulates crown morphology through the canonical Wnt signaling pathway. In addition, while the buccal cusps of *Wnt10a*^*−/−*^ molars were fused together and formed a long crest or ridge, the lingual cusps appeared to be relatively normal, suggesting that there might be a differential expression or functioning of *Wnt10a* on the buccal and lingual sides of the tooth germs. Tabby (*Eda* mutant) and Downless (*Edar* mutant) mice show altered cusp morphology, indicating the significance of EDA-induced NF-*κ*B signaling during tooth morphogenesis (Charles et al. 2009). Along with the findings that *WNT10A* might be a direct target gene of NF-*κ*B signaling (Krappmann et al. 2004) and that crosstalk between the EDA/NF-*κ*B and Wnt/*β*-catenin signaling pathways is important for hair follicle development (Zhang et al. 2009), WNT10A and EDA might be two critical players for crown patterning during odontogenesis. It was recently reported that the morphological complexity of tooth crowns can be controlled by manipulating EDA, Activin, and SHH signaling (Harjunmaa et al. 2012), demonstrating that tooth morphogenesis is an intricate developmental process regulated by many signaling pathways and that tooth shape is a phenotypic trait contributed by many genetic factors.

In addition to abnormal cusp patterning, the molars of *Wnt10a* null mice exhibit taurodontic root morphology, which is also observed in most of the *WNT10A* patients (although this has not previously been reported). During tooth root formation, the cervical loop of the enamel organ forms an epithelial sheet of two cell layers (HERS) that induces the differentiation of root odontoblasts. As the tooth erupts at the same time, the root sheath proliferates and the root elongates (Kumakami-Sakano et al. 2014). Specifically, during root formation of multirooted teeth, the root sheath proliferates toward the center of dental papilla to form tongue-like extensions. When these sheath extensions meet and fuse centrally, the furcation of multirooted teeth forms, as root formation continues apically (Bower 1983). It has been shown that *Wnt10a* is expressed in mature odontoblasts as well as mesenchymal cells of dental papilla adjacent to the root sheath, suggesting a potential role for WNT10A in root development (Yamashiro et al. 2007). Therefore, loss of *WNT10A* function might result in failed or delayed inward extension of HERS, so that a low furcation and an elongated root trunk are formed. Wnt/*β*-catenin signaling has been demonstrated to play critical roles during dental root formation. Both inactivation and overactivation of Wnt/*β*-catenin signaling in dental mesenchyme cause failed or aberrant root development (Bae et al. 2013; Kim et al. 2013), demonstrating that Wnt/*β*-catenin signaling needs to be tightly regulated during root formation. Noticeably, while these mice show severe root defects, *Wnt10a* null mice exhibit root formation with taurodontism, which suggests that other Wnt molecules might also contribute the Wnt/*β*-catenin signaling in dental mesenchyme and that WNT10A is particularly important for the formation of the root furcation in multirooted teeth. Interestingly, it has been reported that taurodontism is a typical feature of teeth from Neanderthals (Kupczik and Hublin 2010). The Neanderthal genome sequence reveals several loss-of-function mutations in *WNT10A* (Prufer et al. 2014), suggesting that *WNT10A* might have contributed to the taurodontic trait of Neanderthals' teeth, although deeper sequencing of more Neanderthal DNA samples is needed to confirm this observation. Interestingly, it has been documented that taurodontism is one of the dental phenotypes in X-linked hypohidrotic ectodermal dysplasia (HED), a syndrome caused by *EDA* mutations. According to one study, 82% (9/11) of HED males and 67% (24/36) of heterozygous female carriers exhibited taurodontic molars (Lexner et al. 2007), which suggested a potential genetic interaction between *WNT10A* and *EDA*. Several mouse models exhibit taurodontism. Conditional depletion of BMP2 in mouse dental mesenchyme results in not only shortened dental roots but also a taurodontic root morphology similar to that of *Wnt10a* null mice (Rakian et al. 2013). Conventional *Evc* knockout mice show abnormal crown morphogenesis as well as root taurodontism (Nakatomi et al. 2013). Moreover, human *distalless homeobox 3* (*DLX3*; OMIM *600525) mutations cause Tricho-Dento-Osseous (TDO) syndrome with taurodontism, while molars manifest the taurodontic phenotype in conditional *Dlx3* knockout mice (Duverger et al. 2012). Research on interactions between these genes would advance our understanding of root development.

Since heterozygous carriers of *WNT10A* mutations were first shown to have missing teeth without apparent other phenotypes of ectodermal dysplasia (Bohring et al. 2009), many different mutations in *WNT10A* have been reported to cause nonsyndromic tooth agenesis with remarkable variation in disease severity. In this study, we reviewed and summarized all the reported *WNT10A* mutations, and noticed a great variability in disease penetrance and expressivity of certain *WNT10A* mutant alleles. For example, p.Phe228Ile mutation was one of the most frequent variants found in patients with *WNT10A* mutations. While some have reported that the heterozygotes of this mutation had more than 10 missing teeth (van den Boogaard et al. 2012), others, including us, found this mutation in cases with only mild tooth agenesis or sometimes in unaffected individuals with a full set of teeth such as the father of Family 2 in this study (Bohring et al. 2009). There are several possibilities to explain this discrepancy. First, because of the high genetic heterogeneity of nonsyndromic tooth agenesis, one can appreciate that this disorder might be digenic or multigenic, which does not follow typical Mendelian inheritance. In other words, sequence variants in more than one gene might contribute to the disease phenotype in a given individual. With different combinations of these sequence variants, one can expect a wide range of disease severity. A study of this multigenic effect in a cohort of 127 probands with nonsyndromic tooth agenesis found that the disease phenotypes in many cases could be better explained by the combined phenotypic effects of alleles in distinct candidate genes (Arte et al. 2013). Another explanation for the discrepancy of the disease severity is the inconclusive (and sometimes suspicious) genotype–phenotype causality in many studies, since for some cases the researcher performed target gene approaches with selected candidate genes and arbitrarily assigned the disease-causing mutations, even if the disease phenotypes were not consistent with what has been reported before. This is particularly true in large cohort studies, where detailed phenotypes and segregation analyses in families are typically absent or incompletely documented.

Therefore, we conclude that for genetic disorders with high genetic heterogeneity, such as nonsyndromic tooth agenesis, detailed documentation and presentation of disease phenotypes, and comprehensive analyses of potential disease-causing sequence variants are the key to conclusive and successful mutational analyses. The genetic causality should not be determined until the disease phenotypes can be well explained by the identified sequence variants. This is particularly important for discerning the disease-causing mutations in sporadic cases, since genetic segregation cannot be confirmed. We believe that with this effort, the phenotypic contribution of sequence variants in specific candidate genes will be clarified and well documented in the literature, which will advance us toward the future of personalized medicine. In this study, we performed whole-exome sequencing in two families and target gene analysis in four families to discern their genetic defects for tooth agenesis. For each case, we scrutinized the phenotypes and identified the mutations that satisfyingly explained the disease severity.

In Family 3, we identified 4 *WNT10A* mutations potentially leading to tooth agenesis in the family, p.Cys107*, p.Gly165Arg, p.Phe228Ile, and p.Asn363His. Interestingly, although of all the recruited family members were compound heterozygous for two of the four mutations, the disease severity of each individual varied significantly. The proband who carried p.Cys107* and p.Phe228Ile mutations had the most severe phenotype, with 23 missing teeth. The p.Cys107* and p.Asn363His compound heterozygote had six permanent teeth missing, whereas the p.Gly165Arg and p.Phe228Ile had two missing teeth. Noticeably, two of the proband's unaffected siblings were both compound heterozygous for p.Gly165Arg and p.Asn363His. This finding suggested that p.Gly165Arg and p.Asn363His might be two rare polymorphisms that do not contribute much to the tooth agenesis phenotype, which is consistent with a previous report (Bohring et al. 2009). The p.Gly165Arg mutation is predicted to be benign with a score of 0.106 by PolyPhen-2, because the substituted Gly^165^ is not highly conserved. In contrast, p.Asn363His changed a highly conserved residue, Asn^363^, and was predicted to be probably damaging with a score of 1.000. However, this sequence variant seemed not to cause very severe phenotypes when combined with p.Cys107* in the father of Family 3. Also, a heterozygous carrier of p.Asn363His did not have missing teeth (Bohring et al. 2009). More cases with this sequence variation need to be characterized to clarify its contribution to the dental phenotype.

We conclude that WNT10A plays significant roles in tooth initiation and crown and root morphogenesis. The dental phenotype observed in *Wnt10a* null mice includes mandibular fourth molars (supernumerary teeth), smaller molars, misshapened crowns and roots (taurodontism), pulp stones, and a susceptibility to root resorption. We identified *WNT10A* defects in six families with nonsyndromic tooth agenesis, including the novel *WNT10A* mutation (g.6825C>T, c.310C>T, p.Arg104Cys). We ruled out defects in other candidate genes for tooth agenesis. Careful inspection of the dental phenotypes in these families identified many instances of misshapened crowns and root taurodonism. Molar crown dysmorphologies and root taurodontism have not previously been reported in families with *WNT10A* defects, but should be routinely documented in future cases. In our *WNT10A* families, pulp stones were only observed in our oldest proband (age 28; family 6). No root resorption was observed in any of our patients, but in consideration of the mouse *Wnt10a* null phenotype, the presence or absence of these phenotypic features should be included in future clinical reports.

## Supporting Information

**Table S1.** Reported *WNT10A* Mutations. **Table S2.** Primers and PCR conditions used for Sanger sequencing. **Figure S1.** Photographs and radiographs of hemimandibles from wild-type 1331 and *Wnt10a* null 1316 mice at 16 weeks. **Figure S2.** Photographs and radiographs of hemimandibles from *Wnt10a* null 1317 and *Wnt10a* null 1329 mice at 16 weeks. **Figure S3.** Radiographs and chart of dental phenotypes in Family 1. **Figure S4.** Oral photographs and chromatograms of subject I:3 in Family 1. **Figure S5.** Oral photographs and chromatograms of subject II:5 in Family 1. **Figure S6.** Oral photographs and chromatograms of subject II:6 in Family 1. **Figure S7.** Oral photographs and chromatograms of subject III:6, the proband of Family 1. **Figure S8.** Oral photographs and chromatograms of subject III:7 in Family 1.

**Figure S9.** Radiographs and chart of dental phenotypes in Family 2. **Figure S10.** Oral photographs and chromatograms of subject IV:3 in Family 2. **Figure S11.** Oral photographs and chromatograms of subject IV:4 (the proband) in Family 2. **Figure S12.** Oral photographs and chromatograms of subject IV:5 in Family 2. **Figure S13.** Radiographs, pedigree, and chart of dental phenotypes in Family 3. **Figure S14.** Chromatograms of Family 3 members II:3, II:4, and III:1. **Figure S15.** Radiograph, chart, oral photographs, pedigree, and sequencing chromatogram of the proband (II-1) in Family 4. **Figure S16.** Radiograph, chart, sequencing chromatograms, pedigree, and oral photographs of subject (II-1), the proband of Family 5. **Figure S17.** Chromatograms of Family 5 members I:1, I:2, and II:2. **Figure S18.** Radiograph, chart, oral photograph, pedigree, and sequencing chromatogram of subject (II-1), the proband of Family 6.
