# Supplementary material for: Taurodontism, variations in tooth number, and misshapened crowns in Wnt10a null mice and human kindreds
Source: Mol Genet Genomic Med. 2014 Sep 15;3(1):40–58. doi: 10.1002/mgg3.111 (PMC4299714; doi:10.1002/mgg3.111)
Supplement: Supplementary file 1 — Table S1. Reported WNT10A Mutations. Table S2. Primers and PCR conditions used for Sanger sequencing. Figure S1. Photographs and radiographs of hemimandibles from wild-type 1331 and Wnt10a null 1316 mice at 16 weeks. Figure S2. Photographs and radiographs of hemimandibles from Wnt10a null 1317 and Wnt10a null 1329 mice at 16 weeks. Figure S3. Radiographs and chart of dental phenotypes in Family 1. Figure S4. Oral photographs and chromatograms of subject I:3 in Family 1. Figure S5. Oral photographs and chromatograms of subject II:5 in Family 1. Figure S6. Oral photographs and chromatograms of subject II:6 in Family 1. Figure S7. Oral photographs and chromatograms of subject III:6, the proband of Family 1. Figure S8. Oral photographs and chromatograms of subject III:7 in Family 1. [file mgg30003-0040-sd1.docx]

Supplemental Data Contents

**Table S1.** Reported *WNT10A* Mutations.

**Table S2. Primers and PCR conditions used for Sanger sequencing.**

**Characterization of *Wnt10a* null mice**

**Figure S1.** Photographs and radiographs of hemimandibles from wild-type 1331 and *Wnt10a* null 1316 mice at 16 weeks.

**Figure S2.** Photographs and radiographs of hemimandibles from *Wnt10a* null 1317 and *Wnt10a* null 1329 mice at 16 weeks.

**Characterization of *WNT10A* Families**

**Figure S3.** Radiographs and chart of dental phenotypes in Family 1.

**Figure S4.** Oral photographs and chromatograms of subject I:3 in Family 1.

**Figure S5.** Oral photographs and chromatograms of subject II:5 in Family 1.

**Figure S6.** Oral photographs and chromatograms of subject II:6 in Family 1.

**Figure S7.** Oral photographs and chromatograms of subject III:6, the proband of Family 1.

**Figure S8.** Oral photographs and chromatograms of subject III:7 in Family 1.

**Figure S9.** Radiographs and chart of dental phenotypes in Family 2.

**Figure S10.** Oral photographs and chromatograms of subject IV:3 in Family 2.

**Figure S11.** Oral photographs and chromatograms of subject IV:4 (the proband) in Family 2.

**Figure S12.** Oral photographs and chromatograms of subject IV:5 in Family 2.

**Figure S13.** Radiographs, pedigree and chart of dental phenotypes in Family 3.

**Figure S14.** Chromatograms of Family 3 members II:3, II:4, and III:1.

**Figure S15.** Radiograph, chart, oral photographs, pedigree, and sequencing chromatogram of the proband (II-1) in Family 4.

**Figure S16.** Radiograph, chart, sequencing chromatograms, pedigree, and oral photographs of subject (II-1), the proband of Family 5.

**Figure S17.** Chromatograms of Family 5 members I:1, I:2, and II:2.

**Figure S18.** Radiograph, chart, oral photograph, pedigree and sequencing chromatogram of subject (II-1), the proband of Family 6.

|  | cDNA change | Protein Change | Heterozygous | Homozygous | Compound Hetero. |
| --- | --- | --- | --- | --- | --- |
|  | c.-14_7delinsC | p.? | ([1](#_ENREF_1)) |  |  |
|  | c.1A>T | p.? | ([2](#_ENREF_2)) |  | ([2](#_ENREF_2)) |
|  | c.27G>A | p.Trp9* | ([3](#_ENREF_3), [4](#_ENREF_4)) | ([3](#_ENREF_3)) |  |
|  | c.146dupT | p.Glu52Glyfs*29 | ([5](#_ENREF_5)) |  |  |
|  | c.208C>T | p.Arg70Trp | ([6](#_ENREF_6)) |  | ([7](#_ENREF_7)) |
|  | c.283G>A | p.Glu95Lys | ([2](#_ENREF_2)) | ([2](#_ENREF_2)) | ([8](#_ENREF_8)) |
|  | c.286T>C | p.Cys96Arg |  |  | ([9](#_ENREF_9)) |
|  | **c.310C>T** | **p.Arg104Cys** | **Family 1** |  | **Family 1** |
|  | **c.321C>A** | **p.Cys107*** | ([2-5](#_ENREF_2), [7-14](#_ENREF_7))  **Family 2** | ([2](#_ENREF_2), [3](#_ENREF_3), [8-12](#_ENREF_8), [14](#_ENREF_14)) | ([2](#_ENREF_2), [3](#_ENREF_3), [5](#_ENREF_5), [7-10](#_ENREF_7), [12-14](#_ENREF_12))  **Families 2 & 3** |
|  | c.337C>T | p.Arg113Cys | ([6](#_ENREF_6)) | ([6](#_ENREF_6)) | ([6](#_ENREF_6), [7](#_ENREF_7), [9](#_ENREF_9)) |
|  | c.347T>C | p.Ile116Thr |  |  | ([5](#_ENREF_5)) |
|  | c376G>A | p.Gly126Ser | ([2](#_ENREF_2)) |  | ([2](#_ENREF_2)) |
|  | c.376+1G>A | Splice Donor: p.? | ([15](#_ENREF_15)) | ([16](#_ENREF_16)) |  |
|  | c.382C>T | p.Arg128* | ([10](#_ENREF_10)) |  | ([3](#_ENREF_3), [9](#_ENREF_9), [10](#_ENREF_10)) |
|  | c.383G>A | p.Arg128Gln | ([3](#_ENREF_3), [8](#_ENREF_8)) |  |  |
|  | c.391G>A | p.Ala131Thr | ([17](#_ENREF_17)) | ([17](#_ENREF_17)) |  |
|  | c.392C>T | p.Ala131Val | ([18](#_ENREF_18)) | ([18](#_ENREF_18)) |  |
|  | c.404C>A | p.Ala135Asp |  |  | ([9](#_ENREF_9)) |
|  | c.409G>A | p.Ala137Thr | ([19](#_ENREF_19)) |  |  |
|  | c.425T>C | p.Val142Ala | ([19](#_ENREF_19)) |  |  |
|  | c.427C>T | p.His143Tyr | ([5](#_ENREF_5)) |  |  |
|  | c.433G>A | p.Val145Met | ([4](#_ENREF_4), [6](#_ENREF_6)) | ([5](#_ENREF_5), [8](#_ENREF_8)) |  |
|  | c.433G>T | p.Val145Leu | ([6](#_ENREF_6)) |  | ([6](#_ENREF_6)) bigenic, *EDARADD* |
|  | c.460C>A | p.Leu154Met | ([6](#_ENREF_6), [19](#_ENREF_19)) |  |  |
|  | c.461T>C | p.Leu154Pro | ([19](#_ENREF_19)) |  |  |
|  | c.487C>T | p.Arg163Trp | ([2](#_ENREF_2), [8](#_ENREF_8)) |  | ([2](#_ENREF_2)) |
|  | **c.493G>A** | **p.Gly165Arg** | ([6](#_ENREF_6)) |  | ([3](#_ENREF_3)) **Family 3** |
|  | c.497 A>G | p.Asp166Gly | ([19](#_ENREF_19)) no phenotype |  |  |
|  | c.503A>T | p.Glu168Val | ([19](#_ENREF_19)) |  |  |
|  | c.511C>T | p.Arg171Cys | ([4](#_ENREF_4), [9](#_ENREF_9), [15](#_ENREF_15), [19](#_ENREF_19)) | ([19](#_ENREF_19)) | ([19](#_ENREF_19), [20](#_ENREF_20)) bigenic, *EDA* |
|  | c.519G>T | p.Lys173Asn | ([19](#_ENREF_19)) |  | ([19](#_ENREF_19)) |
|  | c.545T>C | p.Leu182Pro | ([19](#_ENREF_19)) |  |  |
|  | c.579_592del | pGlu194Alafs*28 | ([6](#_ENREF_6)) |  | ([6](#_ENREF_6)) |
|  | c.622T>C | p.Ser208Pro | ([19](#_ENREF_19)) no phenotype |  |  |
|  | **c.637G>A** | **p.Gly213Ser** | ([4](#_ENREF_4), [7](#_ENREF_7), [15](#_ENREF_15), [19](#_ENREF_19), [20](#_ENREF_20))  **Family 1, 5 & 6** | ([19](#_ENREF_19)) **Family 1** | ([2](#_ENREF_2), [16](#_ENREF_16), [19](#_ENREF_19)) **Family 5**  ([20](#_ENREF_20)) bigenic, *EDA* |
|  | c.649G>A | p.Asp217Asn | ([21](#_ENREF_21)) |  | ([21](#_ENREF_21)) |
|  | c.664G>T | p.Glu222* | ([6](#_ENREF_6)) |  | ([6](#_ENREF_6)) |
|  | c.667C>T | p.Arg223Cys | ([7](#_ENREF_7)) |  |  |
|  | **c.682T>A** | **p.Phe228Ile** | ([2-9](#_ENREF_2), [14](#_ENREF_14), [21](#_ENREF_21))  **Families 2 & 5** | ([2-9](#_ENREF_2))  **Family 4** | ([2](#_ENREF_2), [3](#_ENREF_3), [5-9](#_ENREF_5), [12](#_ENREF_12), [14](#_ENREF_14))  **Families 3 & 5** |
|  | c.694C>T | p.Arg232Trp | ([19](#_ENREF_19)) |  |  |
|  | c.697G>T | p.Glu233* | ([22](#_ENREF_22)) | ([22](#_ENREF_22)) |  |
|  | c.742C>T | p.Arg248* |  | ([5](#_ENREF_5)) |  |
|  | c.796G>T | p.Gly266Cys | ([17](#_ENREF_17)) | ([17](#_ENREF_17)) |  |
|  | c.796G>A | p.Gly266Ser |  |  | ([2](#_ENREF_2)) |
|  | c.826T>C | p.Cys276Arg | ([19](#_ENREF_19)) |  |  |
|  | *c.831G>Y* | p.Trp277Cys |  | ([4](#_ENREF_4)) | ([8](#_ENREF_8)) |
|  | c.862_863insG | p.Ala288Glyfs*140 | ([19](#_ENREF_19)) |  | ([19](#_ENREF_19)) |
|  | c.874A>G | p.Ser292Gly | ([19](#_ENREF_19)) no phenotype |  |  |
|  | c.889G>A | p.Ala297Thr | ([19](#_ENREF_19)) |  |  |
|  | c.895C>T | p.Leu299Phe | ([19](#_ENREF_19)) |  |  |
|  | *c.918C>R* | p.Asn306Lys | ([8](#_ENREF_8)) |  |  |
|  | c.938G>A | p.Gly313Asp | ([19](#_ENREF_19)) |  |  |
|  | *c.945G>H* | p.Ala315Ala | ([9](#_ENREF_9)) |  |  |
|  | c.998C>G | p.Pro333Arg | ([19](#_ENREF_19)) no phenotype |  |  |
|  | c.1016T>G | p.Phe339Cys | ([19](#_ENREF_19)) |  |  |
|  | *c.1036delT* | *p.Cys346Alafs*52* | ([4](#_ENREF_4)) |  |  |
|  | c.1039G>T | p.Glu347* | ([19](#_ENREF_19)) |  | ([16](#_ENREF_16)) |
|  | c.1045G>A | p.Glu349Lys | ([19](#_ENREF_19)) |  |  |
|  | c.1052_1053del | *p.Arg351Profs*76* | ([19](#_ENREF_19)) |  |  |
|  | c.1066G>T | p.Gly356Cys |  | ([16](#_ENREF_16)) |  |
|  | c.1070C>T | p.Thr357Ile | ([19](#_ENREF_19)) |  | ([2](#_ENREF_2)) |
|  | c.1078C>T | p.Arg360Cys | ([2](#_ENREF_2), [5](#_ENREF_5)) |  | ([5](#_ENREF_5)) |
|  | **c.1087A>C** | **p.Asn363His** | ([3](#_ENREF_3), [4](#_ENREF_4)) |  | **Family 3** |
|  | c.1124T>C | p.Met375Thr |  |  | ([9](#_ENREF_9)) |
|  | c.1128C>A | p.Cys376* | ([3](#_ENREF_3)) | ([3](#_ENREF_3)) |  |
|  | c.1135C>T | p.Arg379Cys | ([2](#_ENREF_2)) |  | ([2](#_ENREF_2)) |
|  | c.1139G>A | p.Gly380Asp | ([19](#_ENREF_19)) |  |  |
|  | c.1226_1230del | p.Ile409Argfs*17 | ([2](#_ENREF_2)) |  | ([2](#_ENREF_2)) |

**Table S1. Reported *WNT10A* Mutations.** The c.DNA changes are numbered with respect to the National Center for Biotechnology Information (NCBI) human *WNT10A* mRNA reference sequence: NM_025216.2, with number 1 being the first nucleotide of the *WNT10A* translation initiation codon in exon 1. Mutations in nucleotides 1 to 113 are in exon 1; nucleotides 114 to 376 in exon 2; 377 to 756 in exon 4; 757 to 1254 in exon 4. Heterozygous indicates that the sequence variation was in one *WNT10A* allele only, with no other mutations identified that might have contributed to the phenotype. Homozygous indicates that the sequence variation was identified in both *WNT10A* alleles. Compound Hetero indicates that the sequence variation was in a person with a different *WNT10A* sequence variation in the other allele that could have contributed to the dental phenotype. *WNT10A* sequence variations identified in unaffected controls are indicated as having no phenotype. *WNT10A* sequence variations that combined with a sequence variation in another gene that might have contributed to the phenotype are indicated as being bigenic. Family designations (bold) refer to the families presented in this study. All cDNA and protein designations were checked online using Mutalyzer 2.0.beta-31 (http://www.lovd.nl/mutalyzer/) and corrected designations are in italics.

**Mutation Table References**

1 Abdalla, E.M., Mostowska, A., Jagodzinski, P.P., Dwidar, K. and Ismail, S.R. (2014) A novel WNT10A mutation causes non-syndromic hypodontia in an Egyptian family. *Arch Oral Biol.*, **59**, 722-728.

2 Plaisancie, J., Bailleul-Forestier, I., Gaston, V., Vaysse, F., Lacombe, D., Holder-Espinasse, M., Abramowicz, M., Coubes, C., Plessis, G., Faivre, L. *et al.* (2013) Mutations in WNT10A are frequently involved in oligodontia associated with minor signs of ectodermal dysplasia. *Am J Med Genet A.*, **161**, 671-678.

3 Bohring, A., Stamm, T., Spaich, C., Haase, C., Spree, K., Hehr, U., Hoffmann, M., Ledig, S., Sel, S., Wieacker, P. *et al.* (2009) WNT10A mutations are a frequent cause of a broad spectrum of ectodermal dysplasias with sex-biased manifestation pattern in heterozygotes. *Am J Hum Genet.*, **85**, 97-105.

4 Mues, G., Bonds, J., Xiang, L., Vieira, A.R., Seymen, F., Klein, O. and D'Souza, R.N. (2014) The WNT10A gene in ectodermal dysplasias and selective tooth agenesis. *Am J Med Genet A*, **3**, 36520.

5 Cluzeau, C., Hadj-Rabia, S., Jambou, M., Mansour, S., Guigue, P., Masmoudi, S., Bal, E., Chassaing, N., Vincent, M.C., Viot, G. *et al.* (2011) Only four genes (EDA1, EDAR, EDARADD, and WNT10A) account for 90% of hypohidrotic/anhidrotic ectodermal dysplasia cases. *Hum Mutat.*, **32**, 70-72.

6 Arte, S., Parmanen, S., Pirinen, S., Alaluusua, S. and Nieminen, P. (2013) Candidate gene analysis of tooth agenesis identifies novel mutations in six genes and suggests significant role for WNT and EDA signaling and allele combinations. *PLoS One.*, **8**, e73705.

7 Arzoo, P.S., Klar, J., Bergendal, B., Norderyd, J. and Dahl, N. (2014) WNT10A mutations account for (1/4) of population-based isolated oligodontia and show phenotypic correlations. *Am J Med Genet A.*, **164**, 353-359.

8 van den Boogaard, M.J., Creton, M., Bronkhorst, Y., van der Hout, A., Hennekam, E., Lindhout, D., Cune, M. and Ploos van Amstel, H.K. (2012) Mutations in WNT10A are present in more than half of isolated hypodontia cases. *J Med Genet.*, **49**, 327-331.

9 Mostowska, A., Biedziak, B., Zadurska, M., Dunin-Wilczynska, I., Lianeri, M. and Jagodzinski, P.P. (2013) Nucleotide variants of genes encoding components of the Wnt signalling pathway and the risk of non-syndromic tooth agenesis. *Clin Genet.*, **84**, 429-440.

10 Van Geel, M., Gattas, M., Kesler, Y., Tong, P., Yan, H., Tran, K., Steijlen, P.M., Murrell, D.F. and Van Steensel, M.A. (2010) Phenotypic variability associated with WNT10A nonsense mutations. *Br J Dermatol.*, **162**, 1403-1406.

11 Nagy, N., Wedgeworth, E., Hamada, T., White, J.M., Hashimoto, T. and McGrath, J.A. (2010) Schopf-Schulz-Passarge syndrome resulting from a homozygous nonsense mutation in WNT10A. *J Dermatol Sci.*, **58**, 220-222.

12 Wedgeworth, E.K., Nagy, N., White, J.M., Pembroke, A.C. and McGrath, J.A. (2011) Intra-familial variability of ectodermal defects associated with WNT10A mutations. *Acta Derm Venereol.*, **91**, 346-347.

13 Petrof, G., Fong, K., Lai-Cheong, J.E., Cockayne, S.E. and McGrath, J.A. (2011) Schopf-Schulz-Passarge syndrome resulting from a homozygous nonsense mutation, p.Cys107X, in WNT10A. *Australas J Dermatol.*, **52**, 224-226.

14 Vink, C.P., Ockeloen, C.W., Ten Kate, S., Koolen, D.A., Ploos van Amstel, J.K., Kuijpers-Jagtman, A.M., van Heumen, C.C., Kleefstra, T. and Carels, C.E. (2014) Variability in dentofacial phenotypes in four families with WNT10A mutations. *Eur J Hum Genet*, **8**, 300.

15 Kantaputra, P., Kaewgahya, M. and Kantaputra, W. (2014) WNT10A mutations also associated with agenesis of the maxillary permanent canines, a separate entity. *Am J Med Genet A.*, **164A**, 360-363.

16 Kantaputra, P., Kaewgahya, M., Jotikasthira, D. and Kantaputra, W. (2014) Tricho-odonto-onycho-dermal dysplasia and WNT10A mutations. *Am J Med Genet A.*, **164**, 1041-1048.

17 Castori, M., Castiglia, D., Brancati, F., Foglio, M., Heath, S., Floriddia, G., Madonna, S., Fischer, J. and Zambruno, G. (2011) Two families confirm Schopf-Schulz-Passarge syndrome as a discrete entity within the WNT10A phenotypic spectrum. *Clin Genet.*, **79**, 92-95.

18 Nawaz, S., Klar, J., Wajid, M., Aslam, M., Tariq, M., Schuster, J., Baig, S.M. and Dahl, N. (2009) WNT10A missense mutation associated with a complete odonto-onycho-dermal dysplasia syndrome. *Eur J Hum Genet.*, **17**, 1600-1605.

19 Song, S., Zhao, R., He, H., Zhang, J., Feng, H. and Lin, L. (2014) WNT10A variants are associated with non-syndromic tooth agenesis in the general population. *Hum Genet.*, **133**, 117-124.

20 He, H., Han, D., Feng, H., Qu, H., Song, S., Bai, B. and Zhang, Z. (2013) Involvement of and Interaction between WNT10A and EDA Mutations in Tooth Agenesis Cases in the Chinese Population. *PLoS One.*, **8**, e80393.

21 Kantaputra, P. and Sripathomsawat, W. (2011) WNT10A and isolated hypodontia. *Am J Med Genet A.*, **155A**, 1119-1122.

22 Adaimy, L., Chouery, E., Megarbane, H., Mroueh, S., Delague, V., Nicolas, E., Belguith, H., de Mazancourt, P. and Megarbane, A. (2007) Mutation in WNT10A is associated with an autosomal recessive ectodermal dysplasia: the odonto-onycho-dermal dysplasia. *Am J Hum Genet.*, **81**, 821-828.

| ***EDA*** | | | | |
| --- | --- | --- | --- | --- |
| **Primer** | **Primer sequence** | **Size (bp)** | **Primer** | **Primer sequence** |
| Ex1F | ATTCTTAGCCTCCCCCTCCT | 1046* | Ex1R | TGGTCCTGCCCTCTAAATTG |
| Ex2F | CCCACCCATCATATCCTGTC | 626 | Ex2R | TGGTCCTCTACAGGCAAGGT |
| Ex3F | TACAGTGGAGGGGAAGATGG | 415 | Ex3R | GGCTGGTTTTGAATTCCTCA |
| Ex4F | CGCATGACTCTTCAACCTCA | 618 | Ex4R | AAAAGAAGGGCAGGGAGAAG |
| Ex5F | TCACCCGAAGTCAGGAGTTT | 826 | Ex5R | TGGAGCTAGATGCTGGGAAT |
| Ex6F | GGGTGCACTCTGACTCTTCC | 333 | Ex6R | GCTGTGAGTGAAAACCGTCA |
| Ex7F | ACAGCTGCACAGTGCTTGAC | 549 | Ex7R | GGCATGATGGAGCAAAGAAT |
| Ex8F | GGCCCCATAACAACAAAGAA | 585 | Ex8R | GCAGGAAGTTAGCCATTGGA |
| Ex9F | ATGCCTGTCACCTGTCCTTT | 640 | Ex9R | GGCTCCATCAACTGTCCTGT |
|  |  |  |  |  |
| ***EDAR*** | | | | |
| **Primer** | **Primer sequence** | **Size (bp)** | **Primer** | **Primer sequence** |
| Ex1F | CACTGGTGGTGTGAGGTGTC | 960 | Ex1R | AACCAAATGTGCAACCAACA |
| Ex2F | AACCCTCTGAGCACGAGAAA | 815 | Ex2R | TCTCCTCATCAACGCACTTG |
| Ex3F | GTGATCAACCAGGAGCCACT | 467 | Ex3R | TCTTGCCATCAATCTCAACG |
| Ex4F | GGGGTTTTGCAGTTAGGACA | 552 | Ex4R | ACACAGAGGCAGAGCCTGAT |
| Ex5F | CAGCCACCAGAGAGAGGAAC | 518 | Ex5R | CCTTCATAGACCTGCCTGGA |
| Ex6F | GGTGGAGCTTCTCTGGATCA | 577 | Ex6R | CCTCCTCAATCCCTTCTTCC |
| Ex7-9F | CAGGGTTCCCTGAAAGCAT | 877 | Ex7-9R | GAGGTGGTGGGGACTGTCT |
| Ex10F | TTCACGGAGCATCCATTGT | 586 | Ex10R | CGTCTTGCAGGAGAGCTGA |
| Ex11F | CACCCAGCTGAGCCTAAGAC | 465 | Ex11R | ATTCTGTTTCCCCACACCTG |
| Ex12F | GGTGGCTTTCTGCATGTTTT | 676 | Ex12R | ACAGGCGAGCATCTGAAAGT |
|  |  |  |  |  |
| ***EDARADD*** | | | | |
| **Primer** | **Primer sequence** | **Size (bp)** | **Primer** | **Primer sequence** |
| Ex1F | GGTAATGATGGGGATCGTTG | 989 | Ex1R | AACAGCTTCGGGTTCTCTCA |
| Ex2F | GGCGCCCACCTAATTTTTAT | 565 | Ex2R | AGCCTGGAGAAACCCAAAGT |
| Ex3F | GCATTGAGGAACTGTGCTGA | 872 | Ex3R | CCAAAGTCCCAGCTGTCTTC |
| Ex4F | GCAGCATGTGACACTGAACC | 478 | Ex4R | TTTGCTCATCCACAGAGTGC |
| Ex5F | TTCTGGGCTCAAGCAATCTT | 990 | Ex5R | CTAAACCTGCCCCATTCAGA |
| Ex6F | AGGCCTCTTGTTGACCTGTG | 540 | Ex6R | GTCCACTGTTCCACGTCCTT |
|  |  |  |  |  |
| ***WNT10A*** | | | | |
| **Primer** | **Primer sequence** | **Size (bp)** | **Primer** | **Primer sequence** |
| Ex1F | CCTCGCGGTAACACATATCC | 930* | Ex1R | TCTACCCCAGCAAGAGCATC |
| Ex2F | GGCAGGATGATTGTGAGGAG | 706 | Ex2R | TGACCCAGGAGTCCAGTTCT |
| Ex3F | TTCCTTGTGCCAGACTCTCC | 593 | Ex3R | CGTGGTCCTCAGAAGAGAGG |
| Ex4F | GCGTTTGCCTCTGTATAATGG | 714* | Ex4R | CCTCTTCCCAAGAGCCAAG |
|  |  |  |  |  |
| *5% DMSO is required. | | | | |

**Table S2. Primers and PCR conditions used for Sanger sequencing.**

Primer sets used for amplification of exons and exon-intron junctions of target genes (*EDA*, *EDAR*, *EDARADD*, *WNT10A*,) are listed. Dimethyl sulfoxide (5% or 10% DMSO) was used in PCR reactions with high GC-content amplicons. PCR amplifications were done using the Platinum® PCR Supermix (11306-016; InvitrogenTM by Life Technologies; Grand Island, NY, U.S.A.). The reactions had a 5 min denaturation at 94 ˚C, followed by 35 cycles each with denaturation at 94 ˚C for 30 sec, primer annealing at 56-58 ˚C for 60 sec, and product extension at 72 ˚C for 90 sec. In the final cycle the 72 ˚C extension was for 7 min. Primer annealing was at 56 ˚C for *EDA*, 57 ˚C for *WNT10A*, and 58 ˚C for *EDAR* and *EDARADD*. PCR amplification products were purified by QIAquick PCR Purification Kit and protocol (28106; Qiagen; Valencia, CA, U.S.A.).


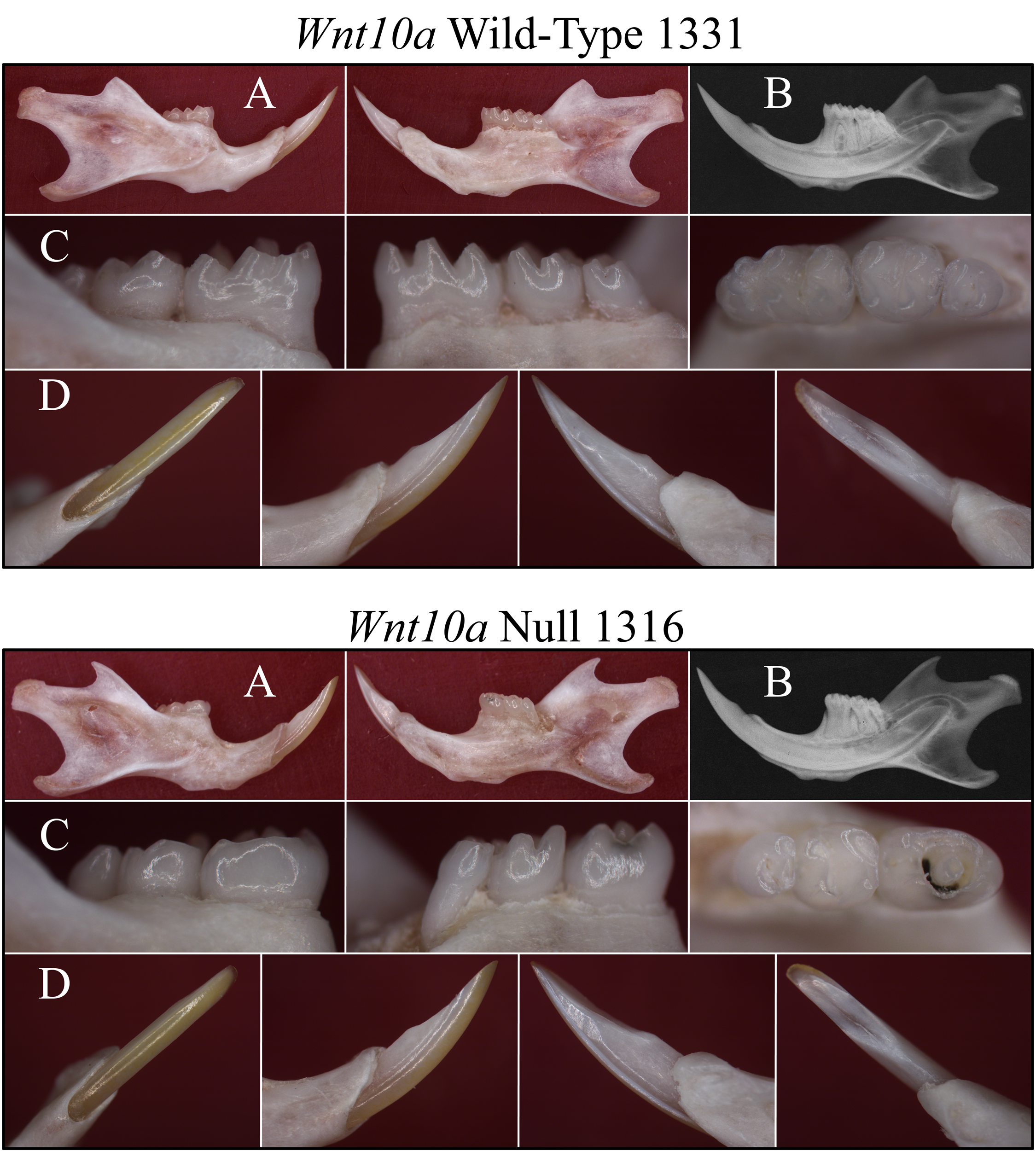


**Figure S1.** Photographs and Radiographs of hemimandibles from wild-type 1331 (top) and *Wnt10a* null 1316 mice (bottom) at 16 weeks. ***A:*** Buccal (left) and lingual (right) aspects of hemimandible. ***B:*** radiograph of hemimandible. ***C:*** (from left to right) buccal, lingual, and occlusal photographs of molars. ***D:*** (from left to right) labial, mesial, distal, lingual photographs of incisor tip.


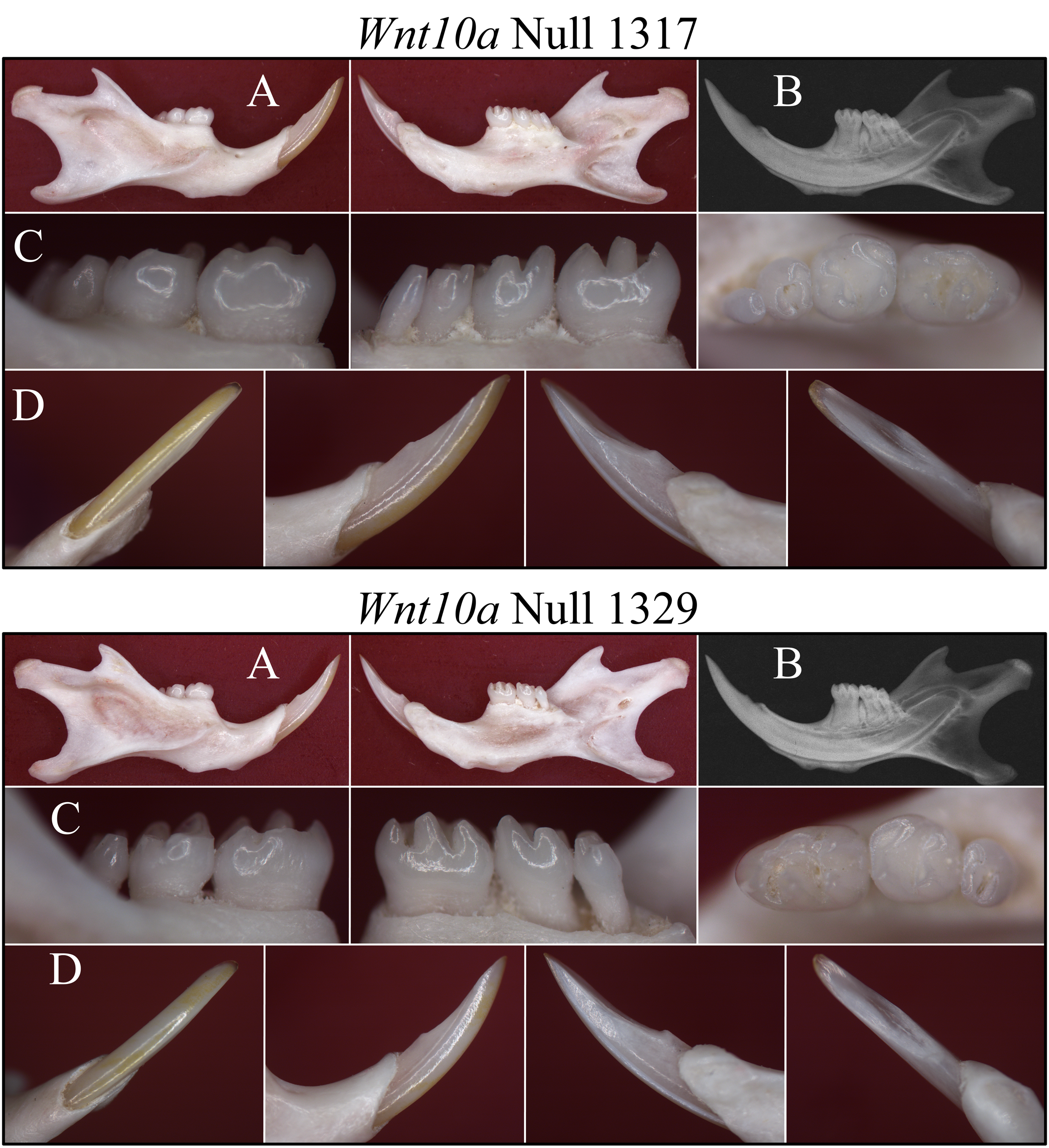


**Figure S2.** Photographs and Radiographs of hemimandibles from *Wnt10a* null 1317 (top) and *Wnt10a* null 1329 mice (bottom) at 16 weeks. ***A:*** Buccal (left) and lingual (right) aspects of hemimandible. ***B:*** radiograph of hemimandible. ***C:*** (from left to right) buccal, lingual, and occlusal photographs of molars. ***D:*** (from left to right) labial, mesial, distal, lingual photographs of incisor tip.


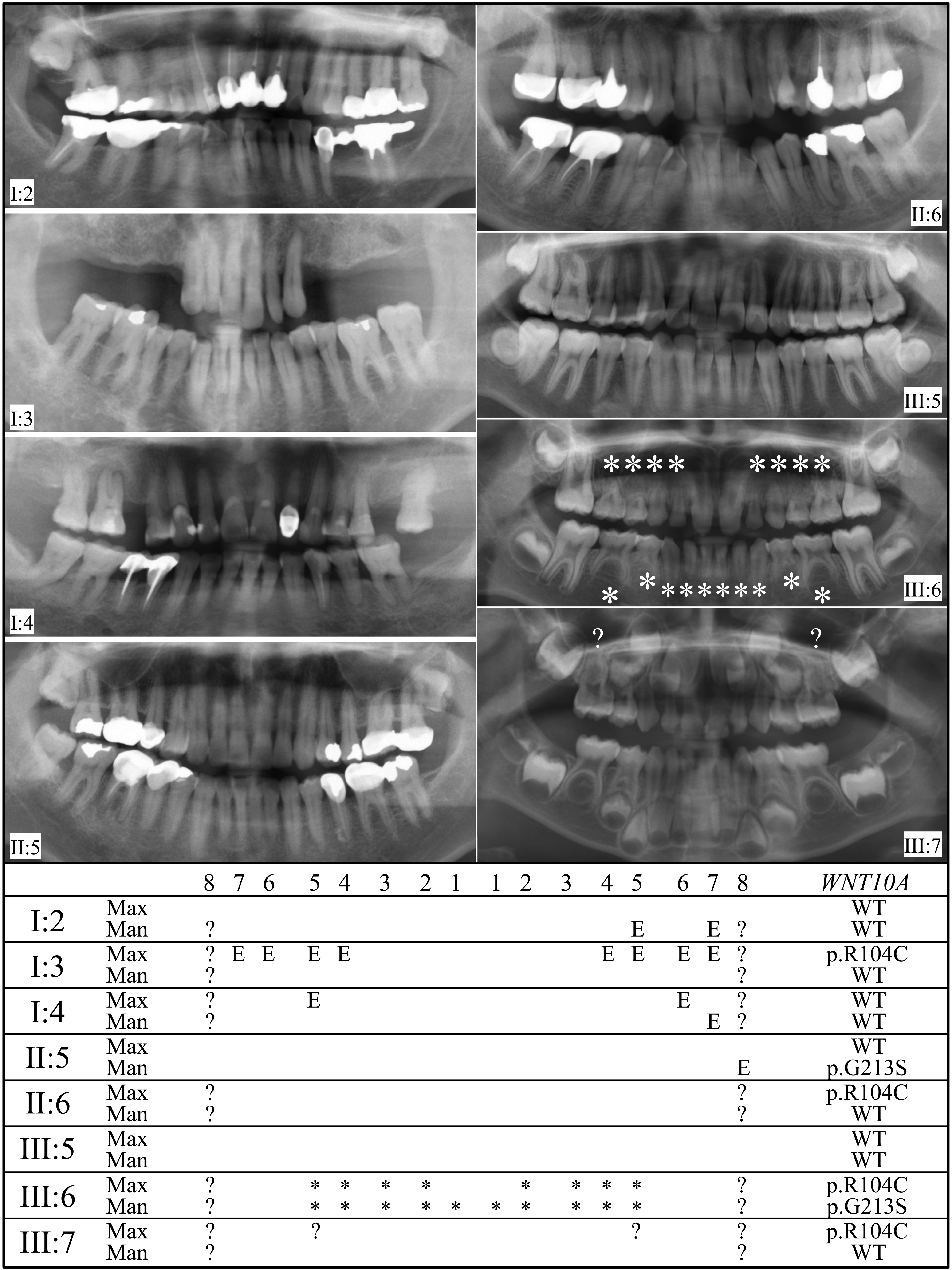


**Figure S3.** Radiographs and chart of dental phenotypes in Family 1. The number in each panorex (top) and at the left in each row of the chart (bottom) corresponds to the individual’s place in the pedigree (shown in Figs. 1 and S4). ***Key:*** *, tooth never formed; E, tooth was extracted; ?, unknown if tooth will form because of age at the time of the radiograph. The subjects ages at the times of the radiographs were: I:2, 69y; I:3, 71y; I:6, 68y; II:5, 49y; II:6, 43y; III:5, 13y7m; III:6, 8y9m; III:7, 4y7m. No other *WNT10A* sequence variations were observed. Further genotype and phenotype information for Family 1 members I:3, II:5, II:6, and III:7 is provided in Figures S4 to S7, respectively.


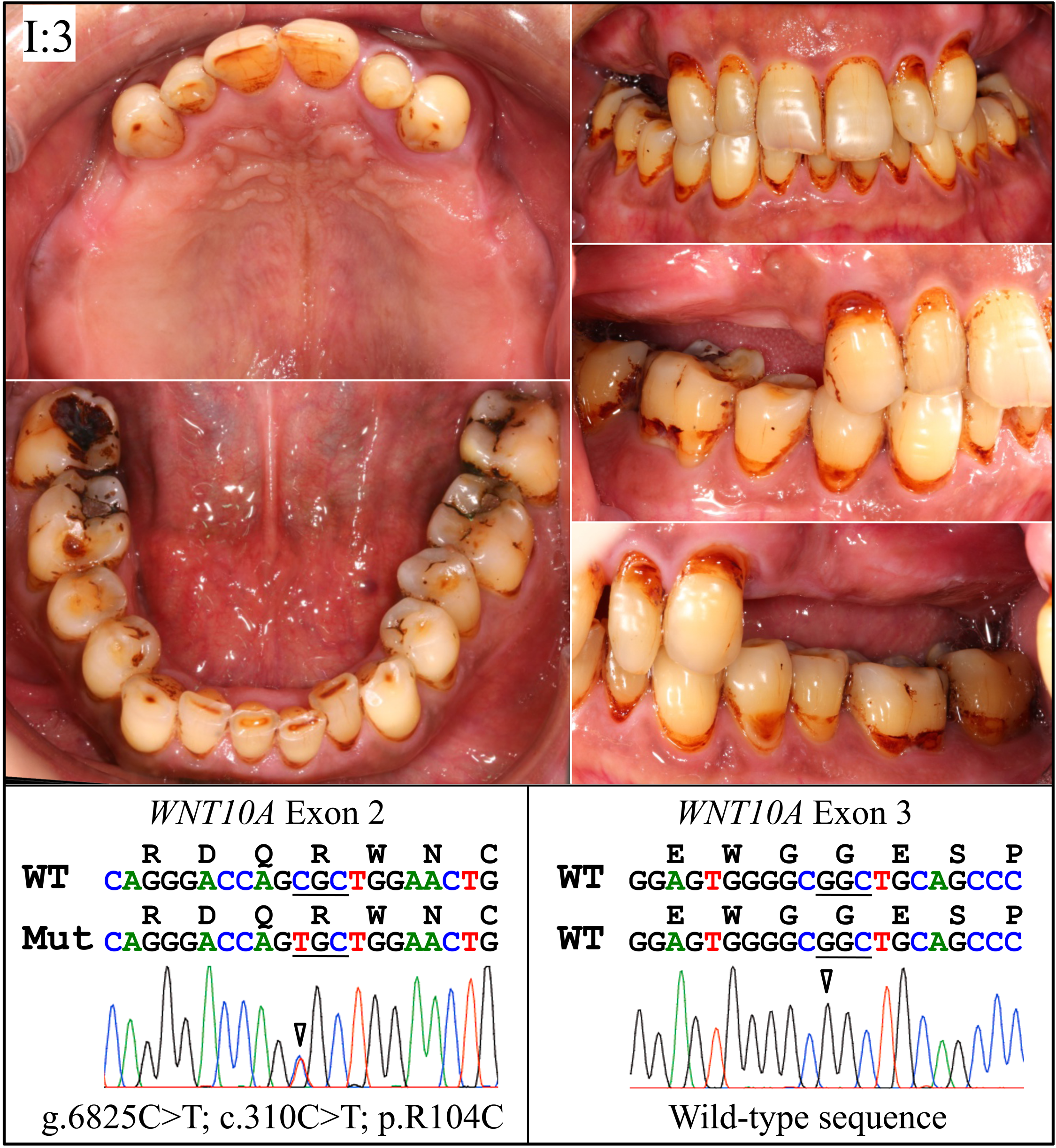


| **Figure S4.** Oral photographs and chromatograms of subject I:3 (age 71y) in Family 1. The chromatogram of *WNT10A* exon 2 sequence shows the proband was heterozygous for the p.Arg104Cys variation (left), but lacked the exon 3 sequence variation (g.14712G>A; c.637G>A; p.Gly213Ser) found in other family members (II:5 and III:6). No other *WNT10A* sequence variations were observed. The only teeth absent from this subject had been extracted. | Family 1 Pedigree  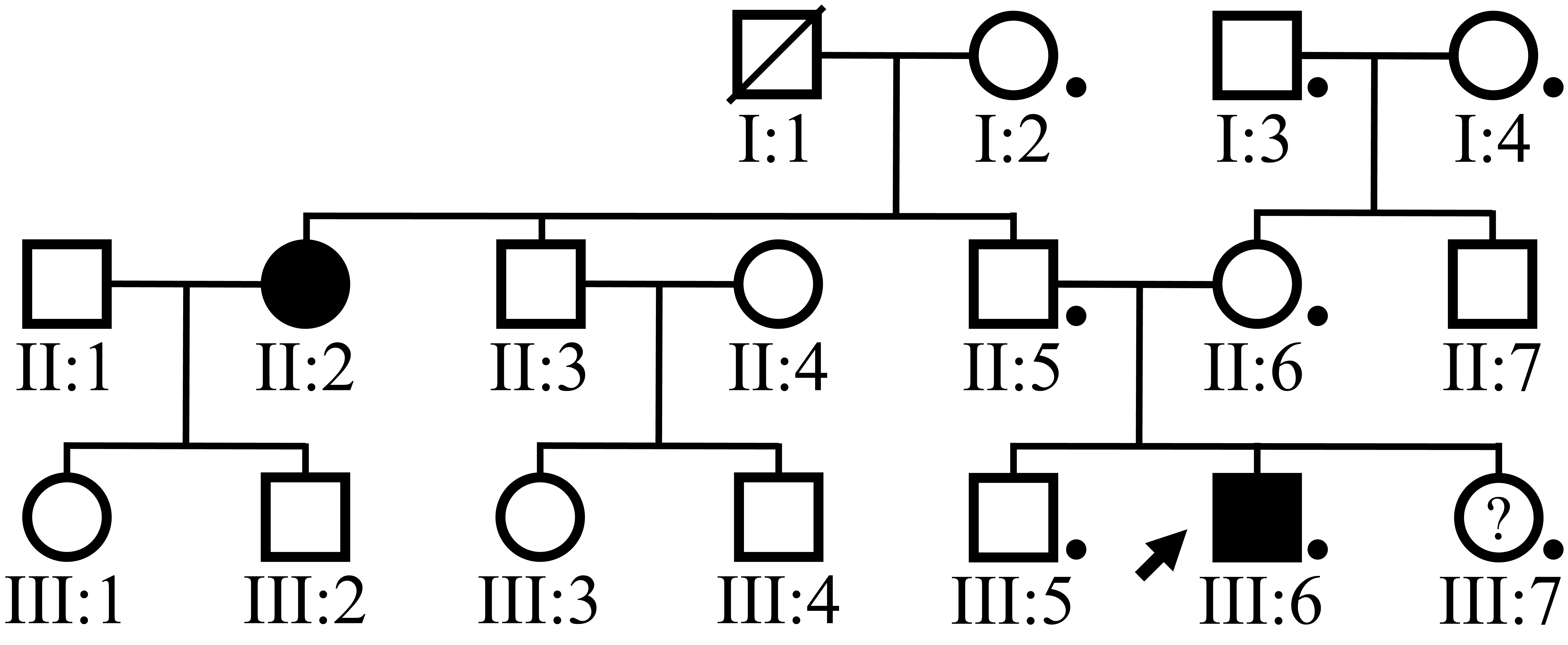 |
| --- | --- |


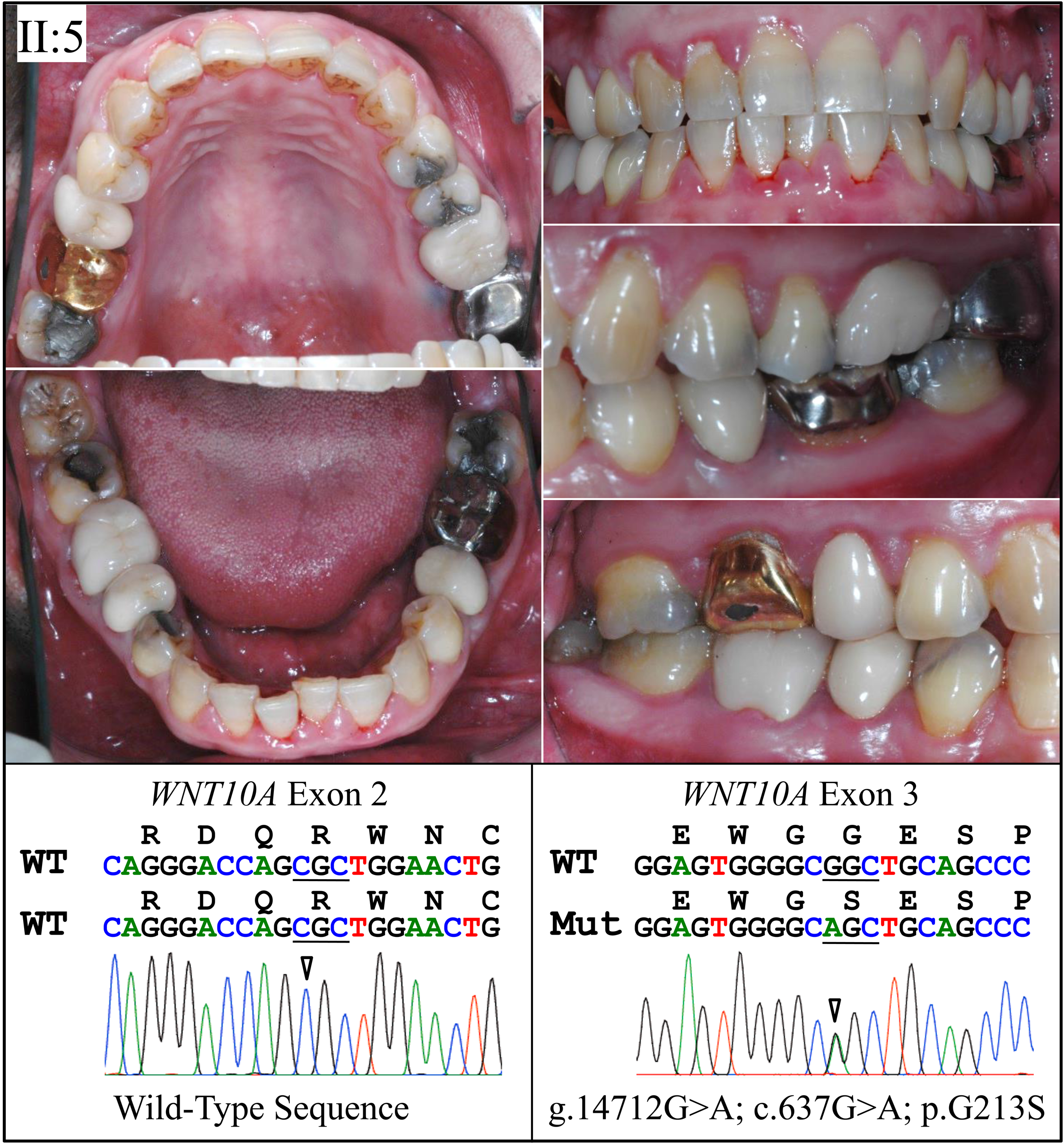


**Figure S5.** Oral photographs and chromatograms of subject II:5 (age 49y) in Family 1. The *WNT10A* exon 2 and exon 3 chromatograms shows that subject II:5 was heterozygous for the p.Gly213Ser variation (right), but lacked the exon 2 sequence variation (g.6825C>T; c.310C>T; p.Arg104Cys) found in other family members (I:3, II:6; III:6 and III:7). No other *WNT10A* sequence variations were observed. No teeth were absent.


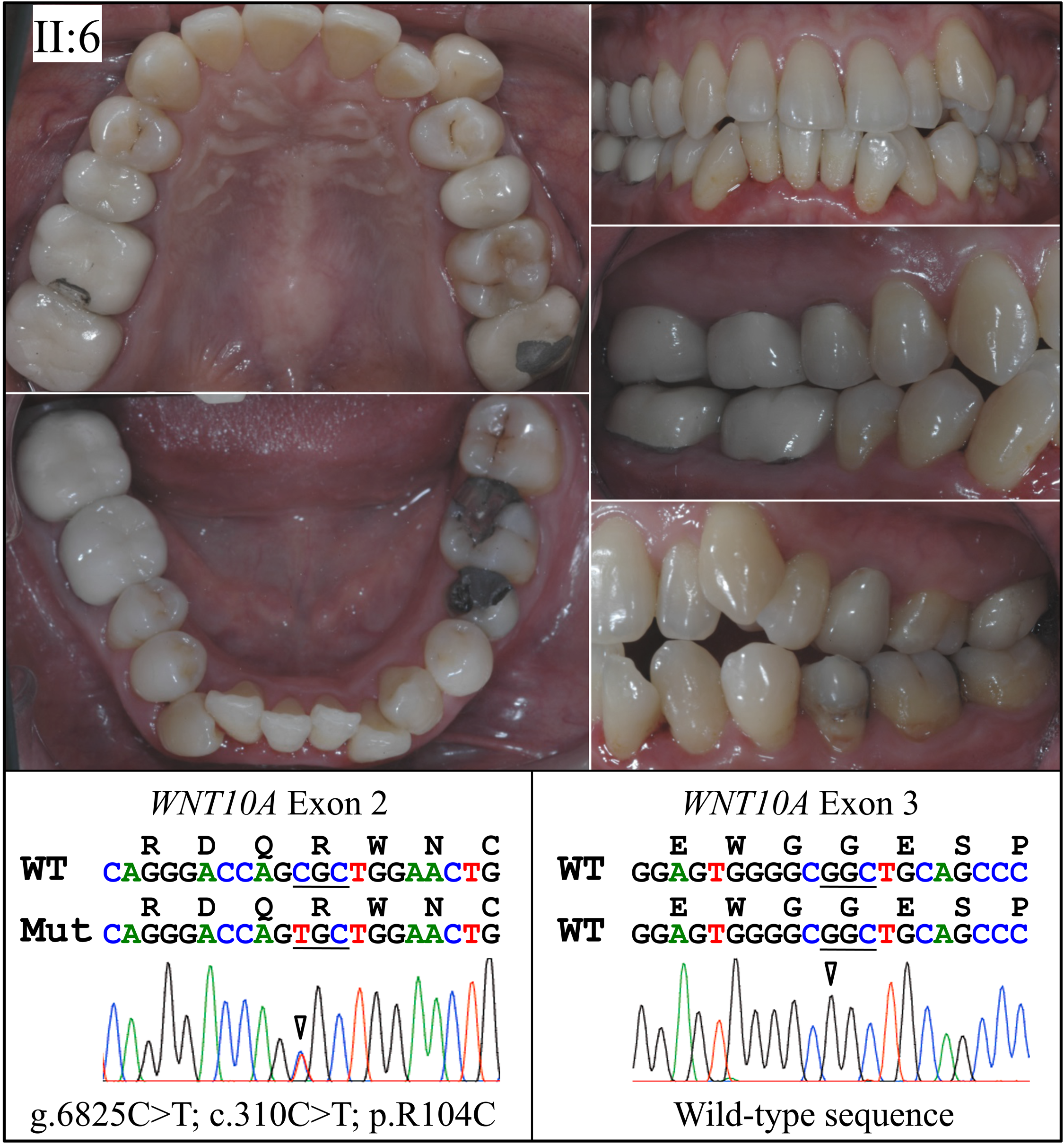


**Figure S6.** Oral photographs and chromatograms of subject II:6 (age 43y) in Family 1. The *WNT10A* exon 2 and exon 3 chromatograms shows that subject II:6 was heterozygous for the exon 2 sequence variation (g.6825C>T; c.310C>T; p.Arg104Cys) but lacked the p.Gly213Ser variation (right) found in other family members (II:5 and III:6). No other *WNT10A* sequence variations were observed. No teeth were absent.


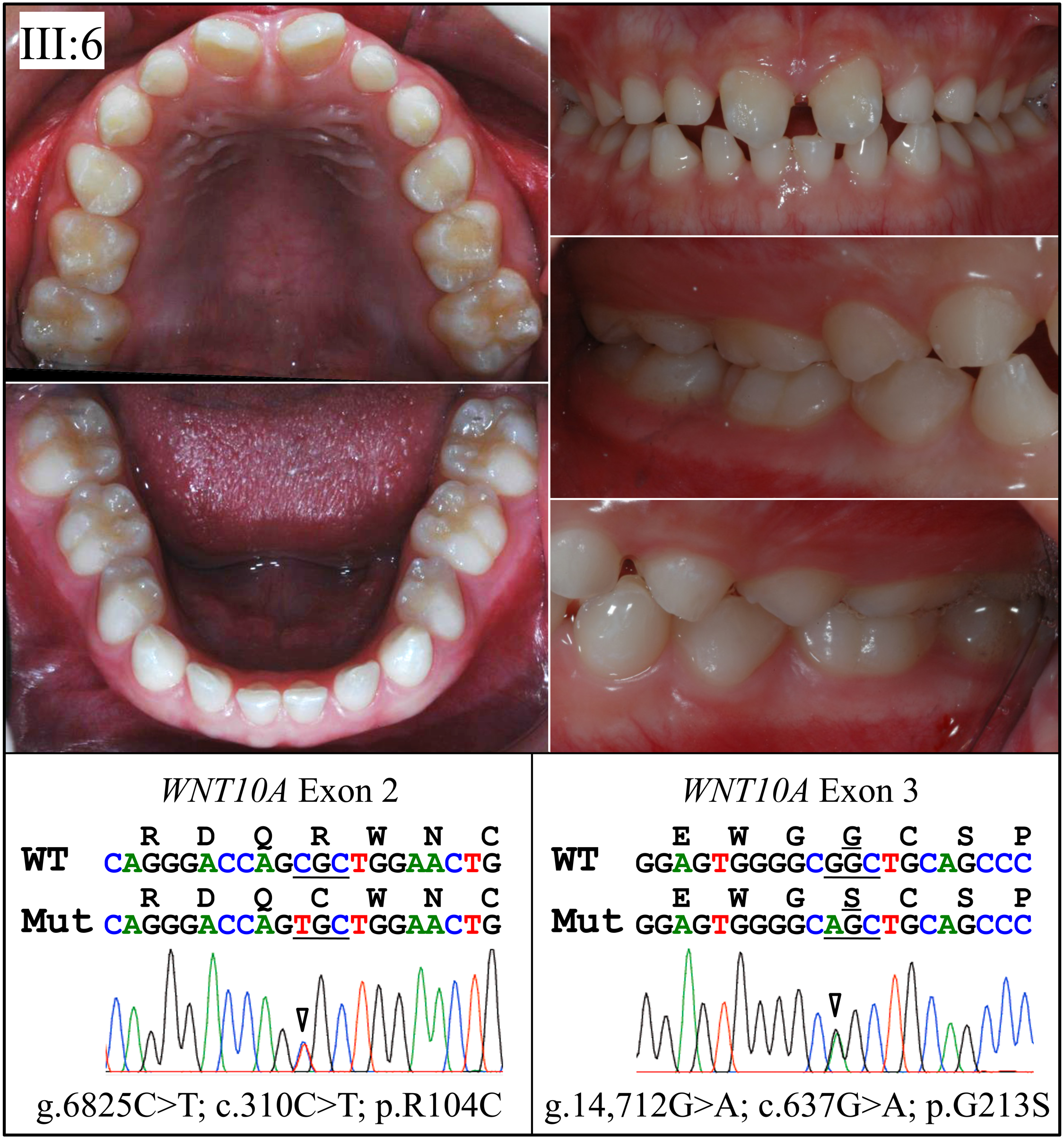


**Figure S7.** Oral photographs and chromatograms of subject III:6 (age 8y9mo), the proband of Family 1. The *WNT10A* exon 2 and exon 3 chromatograms shows that subject III:6 was heterozygous for the exon 2 sequence variation (g.6825C>T; c.310C>T; p.Arg104Cys) and the p.Gly213Ser variation (right). No other *WNT10A* sequence variations were observed. This individual had severe oligodontia, with 18 permanent teeth absent, excluding third molars.


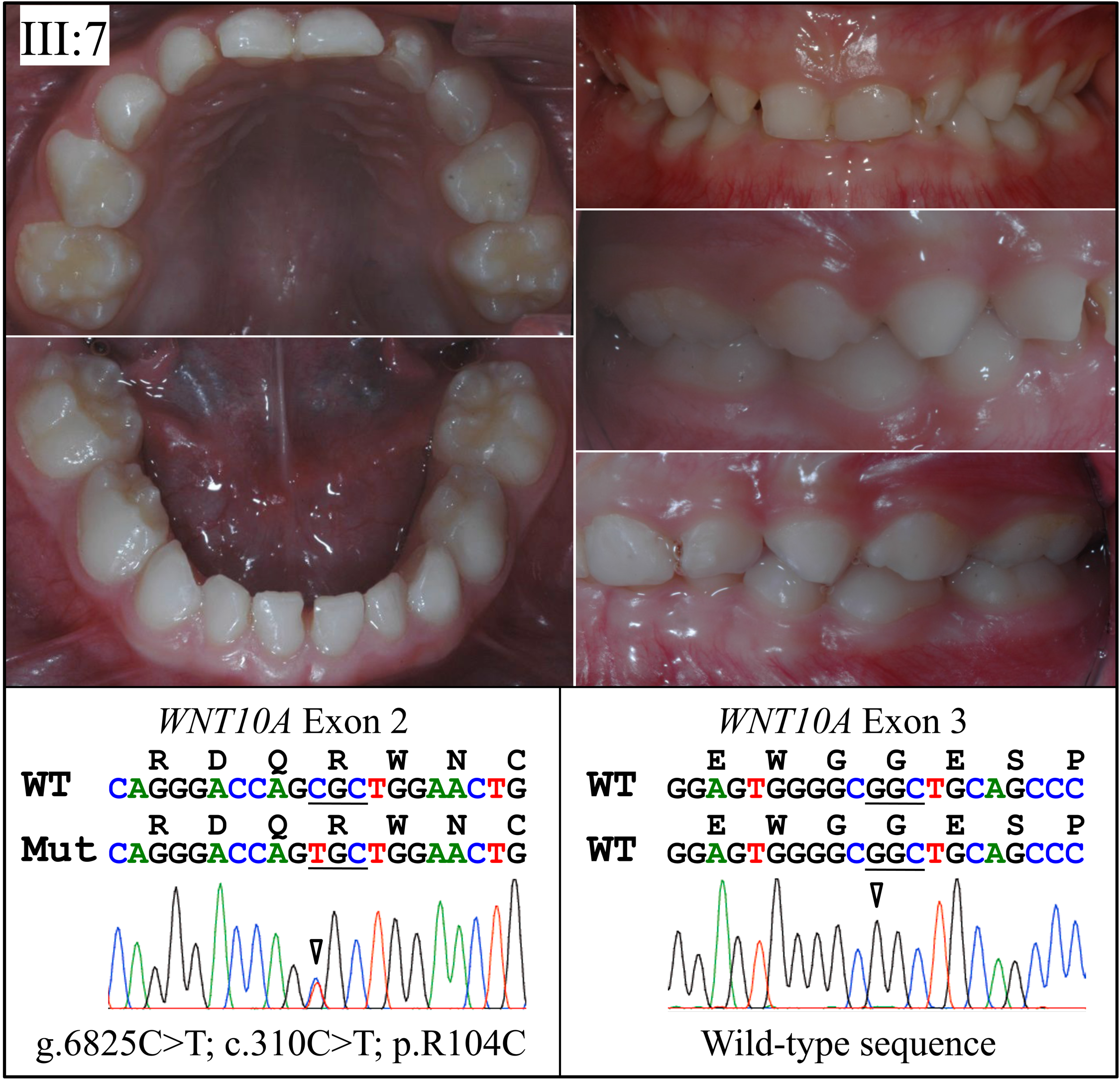


**Figure S8.** Oral photographs and chromatograms of subject III:7 (age 4y7m) in Family 1. The *WNT10A* exon 2 and exon 3 chromatograms shows that subject II:7 was heterozygous for the exon 2 sequence variation (g.6825C>T; c.310C>T; p.Arg104Cys) but lacked the p.Gly213Ser variation (right) found in other family members (II:5 and III:6). No other *WNT10A* sequence variations were observed. No teeth were absent.
