# Supplementary material for: Taurodontism, variations in tooth number, and misshapened crowns in Wnt10a null mice and human kindreds
Source: Mol Genet Genomic Med. 2014 Sep 15;3(1):40–58. doi: 10.1002/mgg3.111 (PMC4299714; doi:10.1002/mgg3.111)
Supplement: Supplementary file 2 — Figure S9. Radiographs and chart of dental phenotypes in Family 2. Figure S10. Oral photographs and chromatograms of subject IV:3 in Family 2. Figure S11. Oral photographs and chromatograms of subject IV:4 (the proband) in Family 2. Figure S12. Oral photographs and chromatograms of subject IV:5 in Family 2. Figure S13. Radiographs, pedigree, and chart of dental phenotypes in Family 3. Figure S14. Chromatograms of Family 3 members II:3, II:4, and III:1. Figure S15. Radiograph, chart, oral photographs, pedigree, and sequencing chromatogram of the proband (II-1) in Family 4. Figure S16. Radiograph, chart, sequencing chromatograms, pedigree, and oral photographs of subject (II-1), the proband of Family 5. Figure S17. Chromatograms of Family 5 members I:1, I:2, and II:2. Figure S18. Radiograph, chart, oral photograph, pedigree, and sequencing chromatogram of subject (II-1), the proband of Family 6. [file mgg30003-0040-sd2.docx]

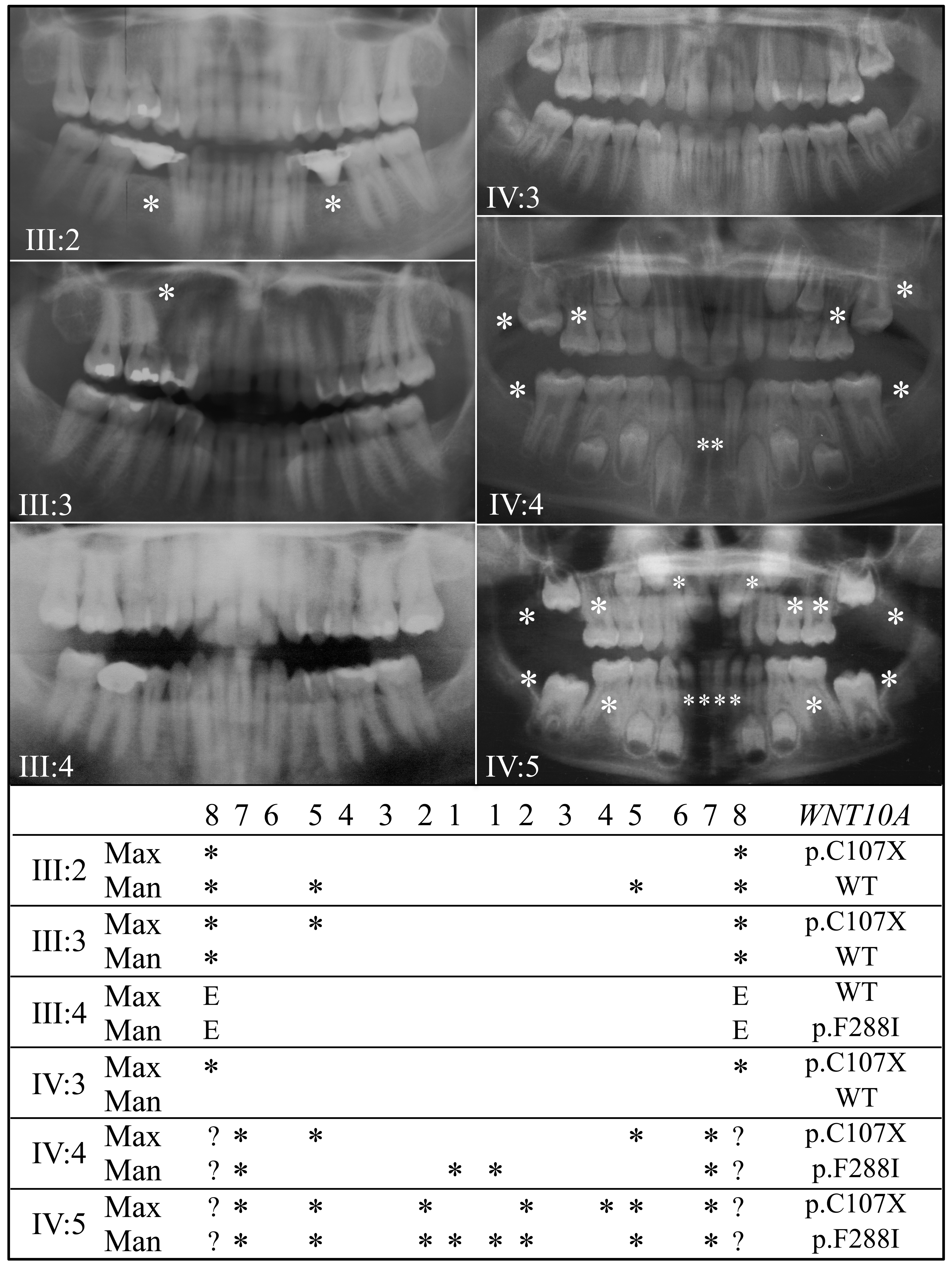


**Figure S9.** Radiographs and chart of dental phenotypes in Family 2. The number in each panorex (top) and at the left in each row of the chart (bottom) corresponds to the individual’s place in the pedigree (Fig. 1). ***Key:*** *, tooth never formed; E, tooth was extracted; ?, unknown if tooth will form because of age at the time of the radiograph. At the times the radiographs were taken, subjects III:2, III:3 and III:4 were mature adults. The young subjects were IV:3 (12y), IV:4 (9y3m) and IV:5 (6y6m) ages. No other *WNT10A* sequence variations were observed.


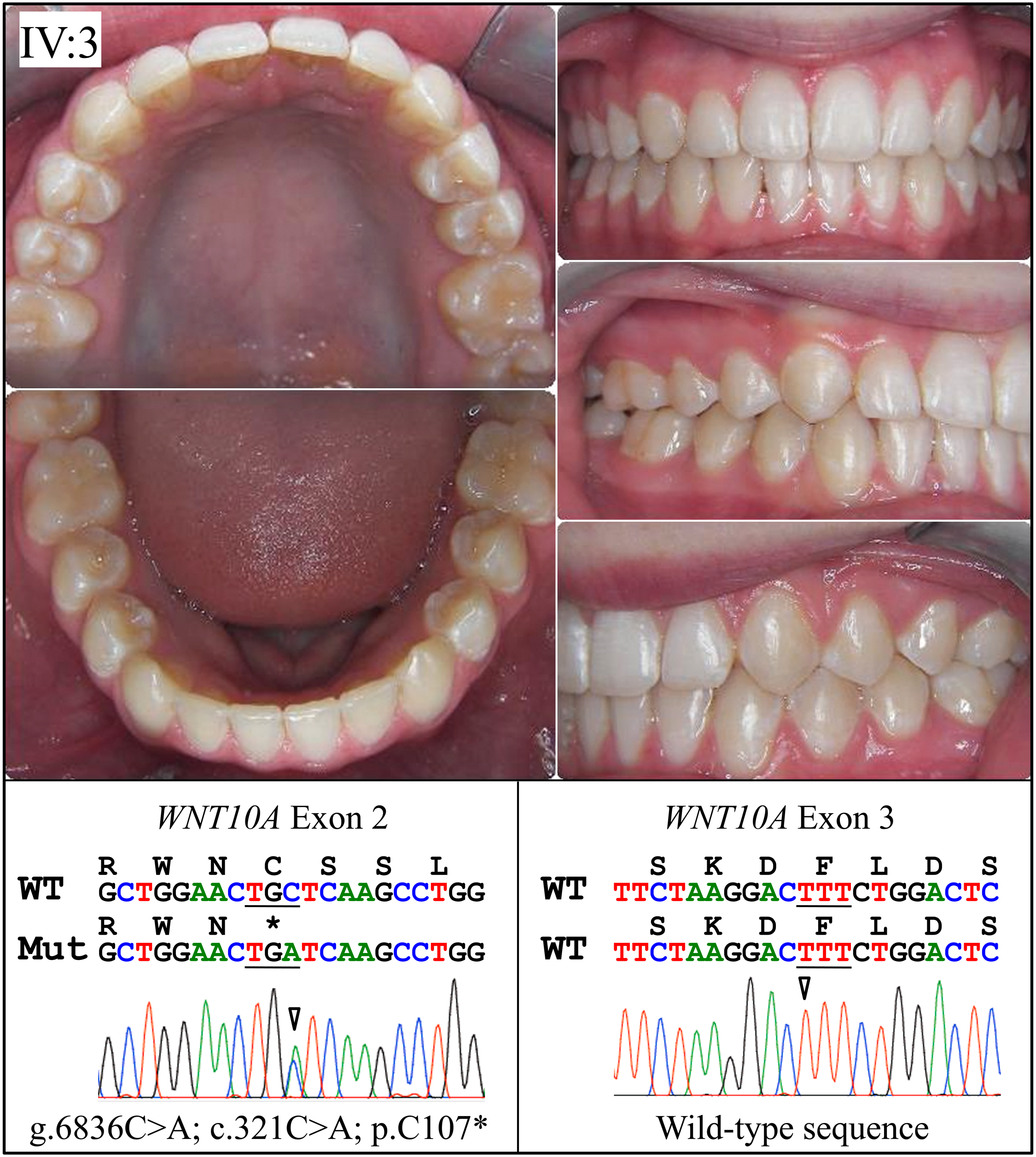


| **Figure S10.** Oral photographs (age 14 years) and chromatograms of subject IV:3 in Family 2. The *WNT10A* exon 2 and exon 3 chromatograms show that subject IV:3 was heterozygous for the exon 2 sequence variation g.6836C>T; c.321C>A; p.Cys107* (left) but lacked the g.14,758T>A; c.682T>A; p.Phe228Ile variation (right) found in family members III:4, IV:4, and IV:5 (pedigree at right). No other *WNT10A* sequence variations were observed. No teeth excepting two 3^rd^ molars were absent. | 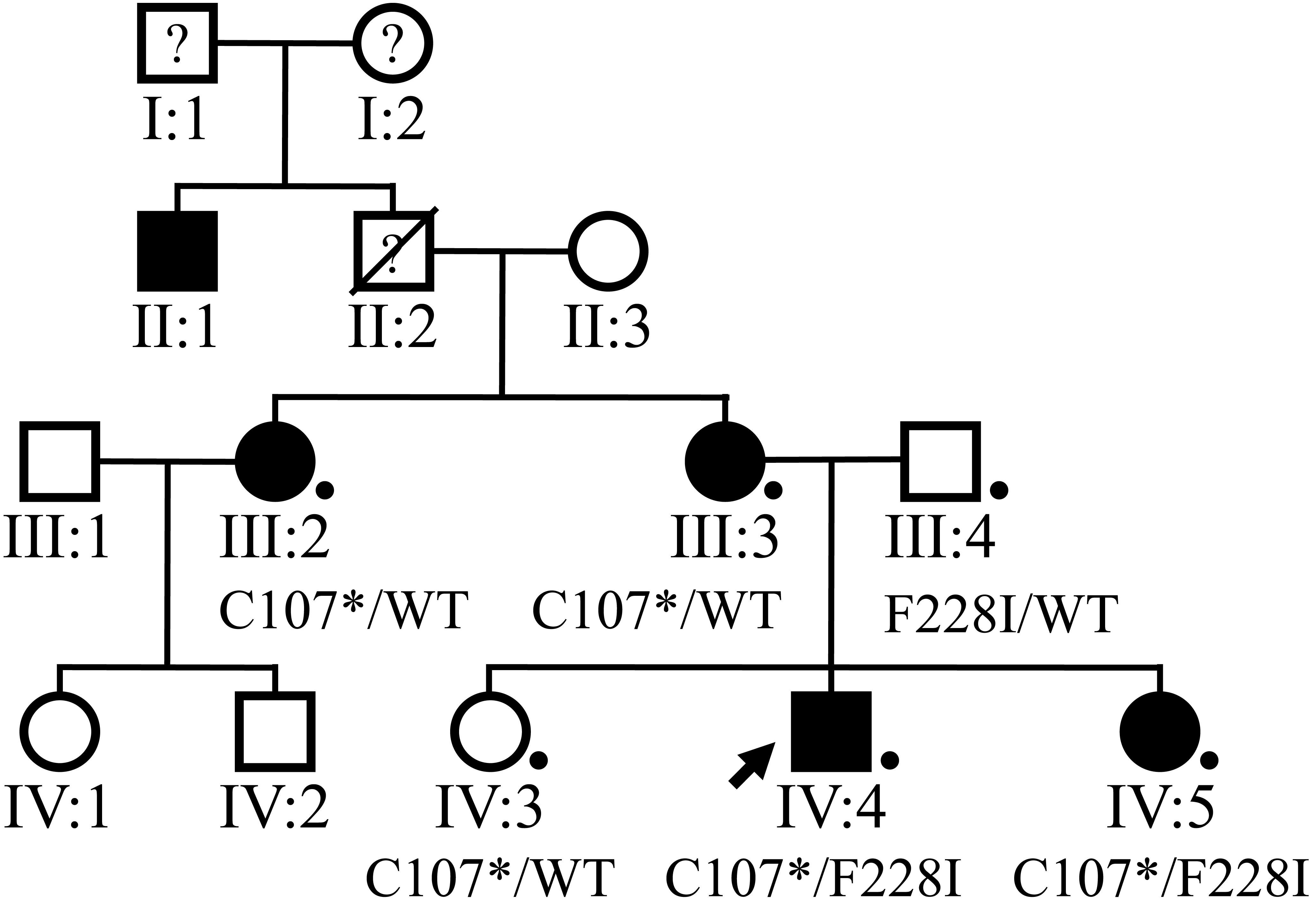 |
| --- | --- |

**
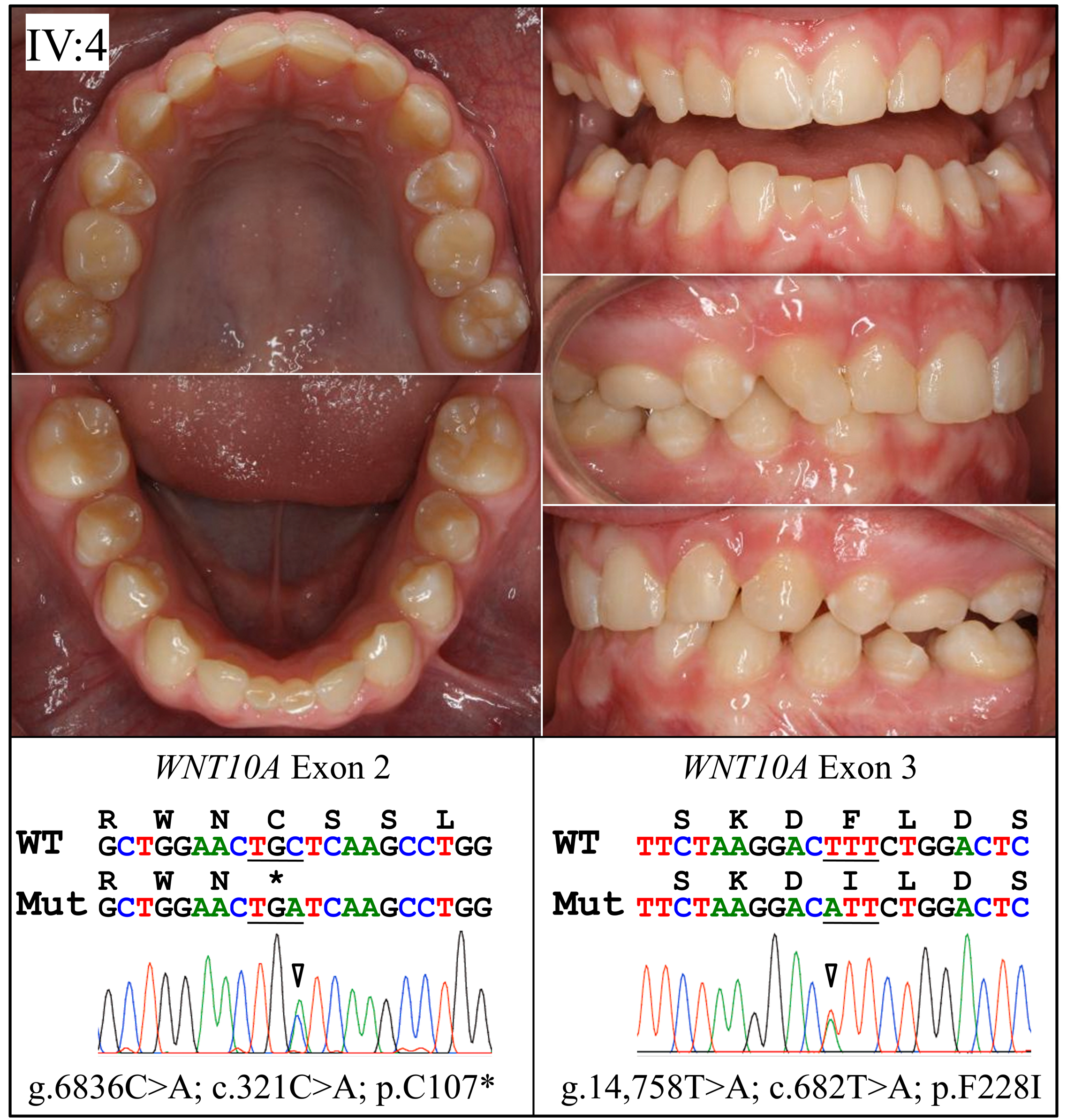
**

**Figure S11.** Oral photographs (at age 13years, 1 month) and chromatograms of subject IV:4 (the proband) in Family 2. The *WNT10A* exon 2 and exon 3 chromatograms show that subject IV:4 was heterozygous for the both the exon 2 sequence variation g.6836C>T; c.321C>A; p.Cys107* (left) as well as the g.14,758T>A; c.682T>A; p.Phe228Ile variation (right). No other *WNT10A* sequence variations were observed. Eight permanent teeth not counting 3^rd^ molars were absent.

**
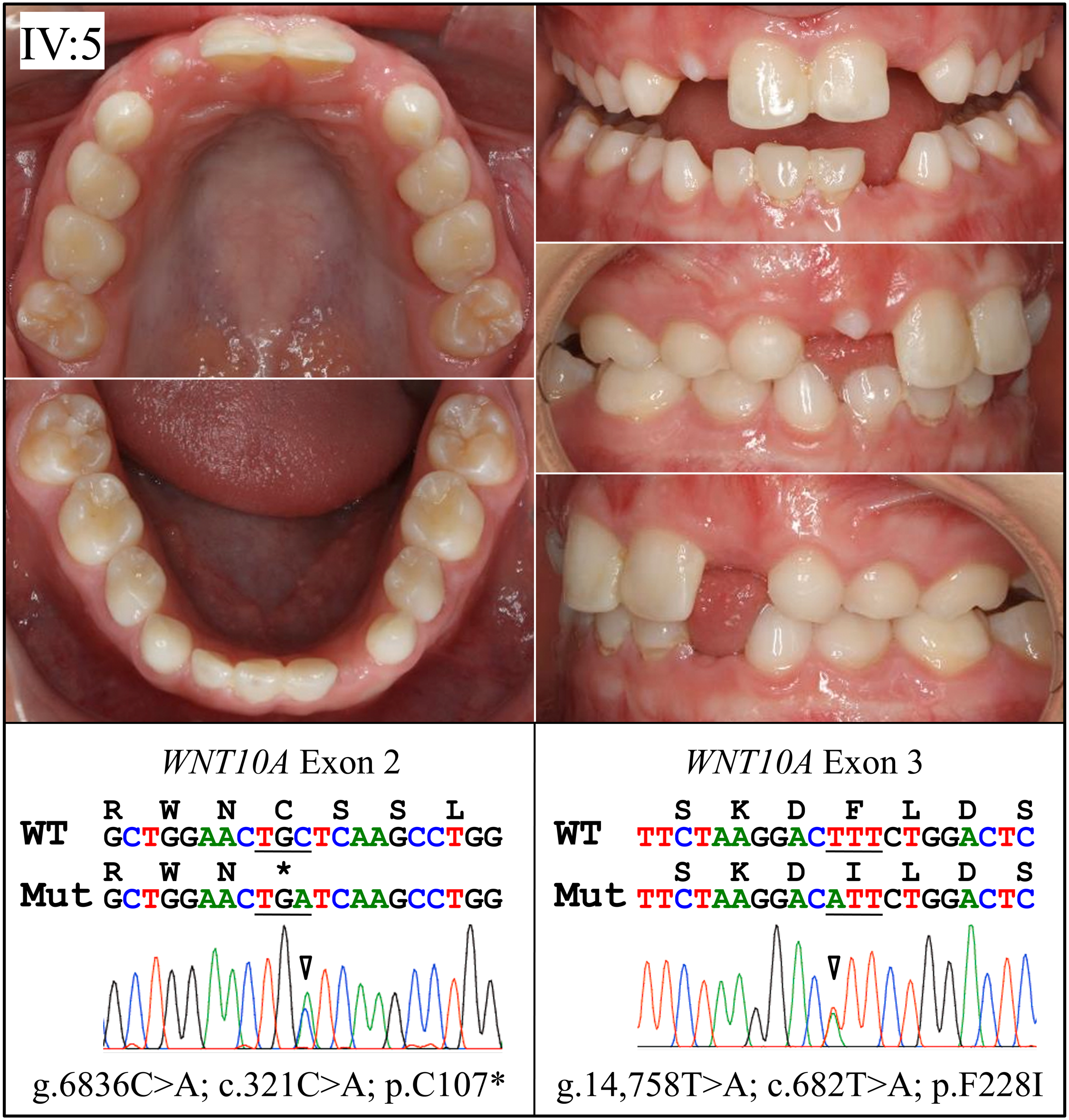
**

**Figure S12.** Oral photographs (at age 10 years, 7 months) and chromatograms of subject IV:5 in Family 2. The *WNT10A* exon 2 and exon 3 chromatograms show that subject IV:5 was heterozygous for the both the exon 2 sequence variation g.6836C>T; c.321C>A; p.Cys107* (left) as well as the g.14,758T>A; c.682T>A; p.Phe228Ile variation (right). No other *WNT10A* sequence variations were observed. Fifteen permanent teeth not counting 3^rd^ molars were absent.


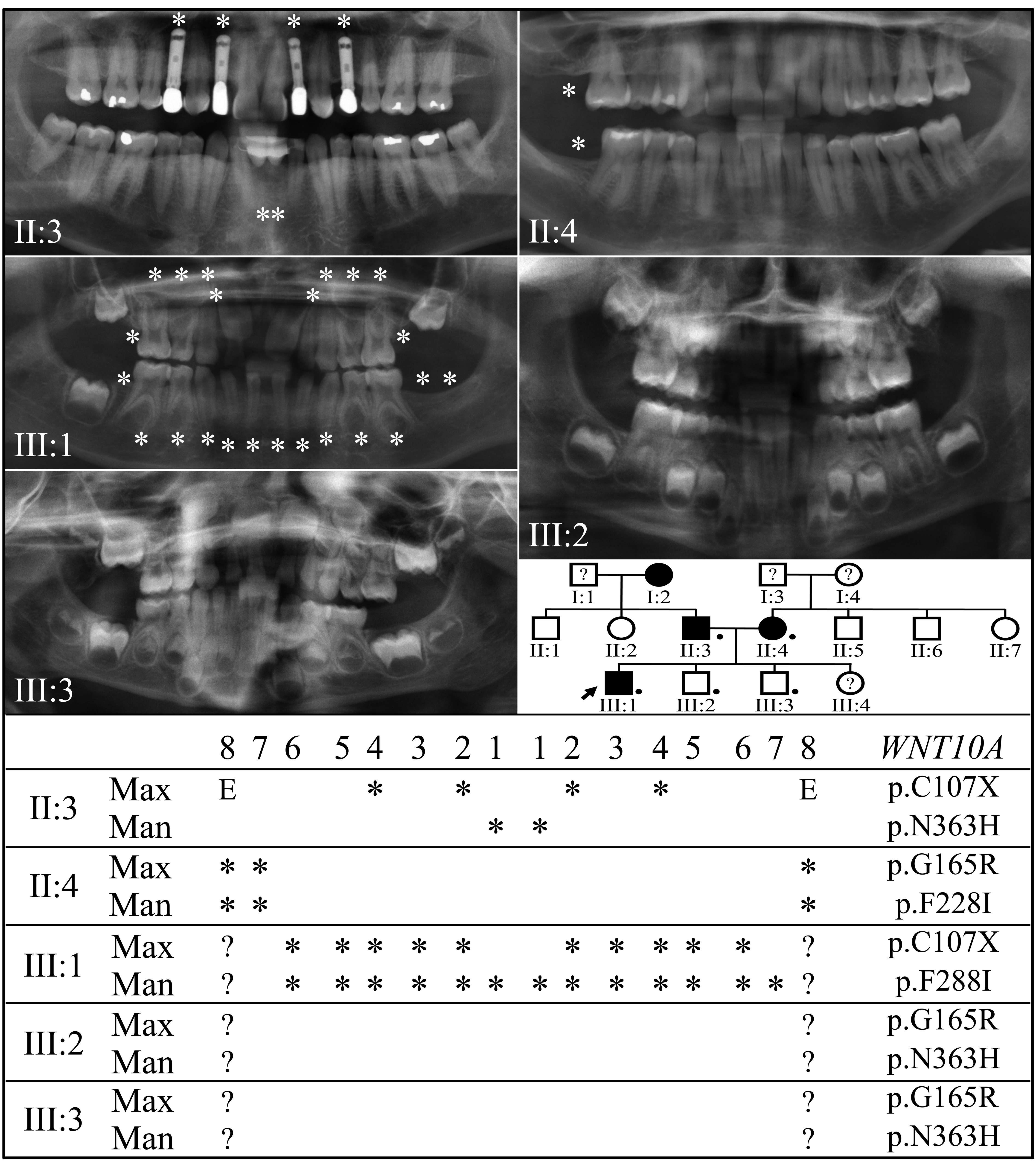


**Figure S13.** Radiographs, pedigree and chart of dental phenotypes in Family 3. The number in each panorex (top) and at the left in each row of the chart (bottom) corresponds to the individual’s place in the pedigree. ***Key:*** *, tooth never formed; E, tooth was extracted; ?, unknown if tooth will form because of age at the time of the radiograph. At the times the radiographs were taken, subjects II:3, and II:4 were mature adults. The young subjects were III:1 (6y), III:2 (6y) and III:3 (3y) ages. No other *WNT10A* sequence variations were observed.


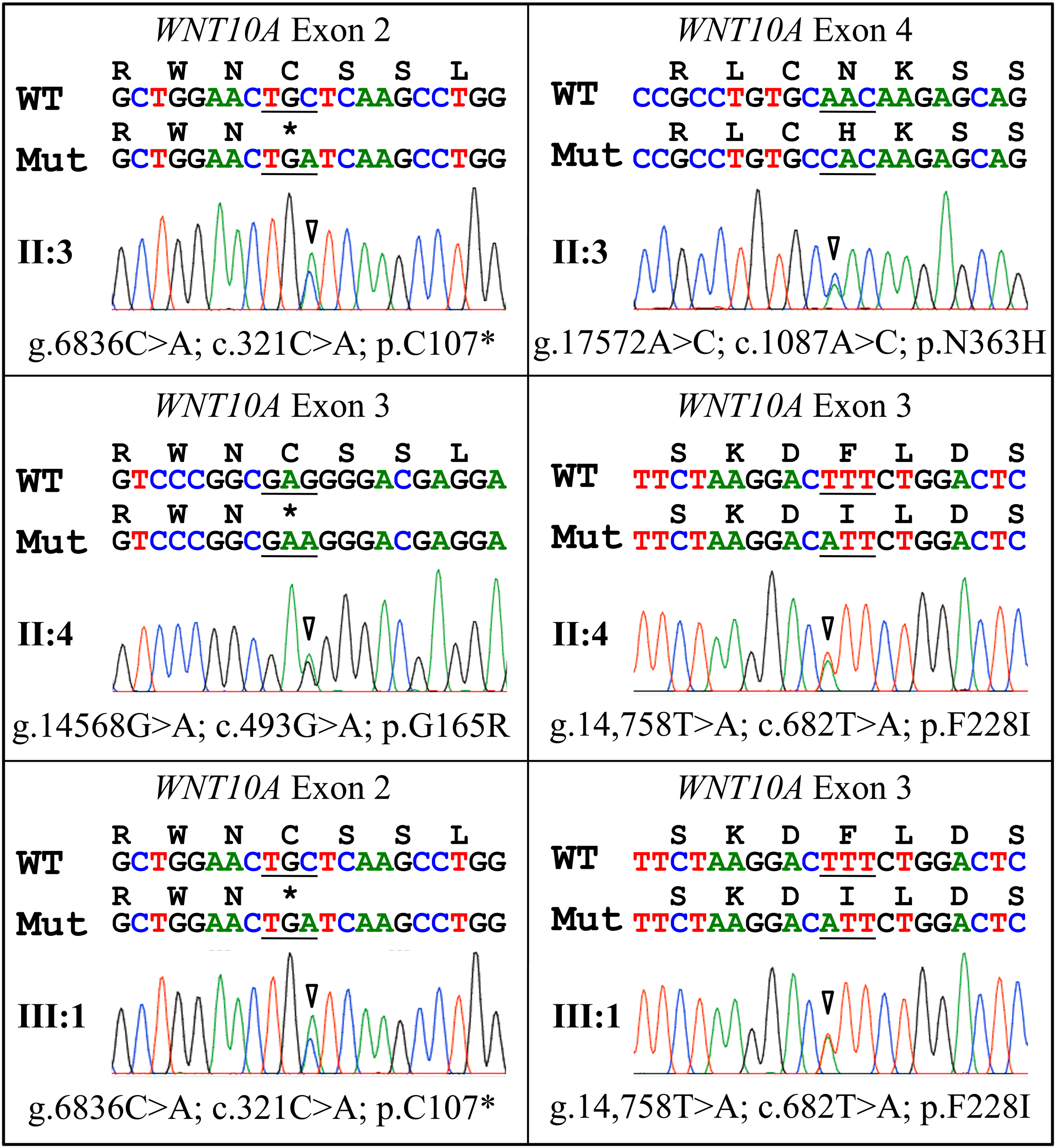


| **Figure S14.** Chromatograms of Family 3 members II:3, II:4, and III:1. Subject II:3 was heterozygous for *WNT10A* mutations pCys107* and pAsn363His and was missing 6 teeth excluding 3^rd^ molars. Subject II:4 was heterozygous for p.Gly165Arg and p.Phe228Ile and was missing 2 teeth excluding 3^rd^ molars. The proband (III:1) was missing 23 permanent teeth excluding 3^rd^ molars. | **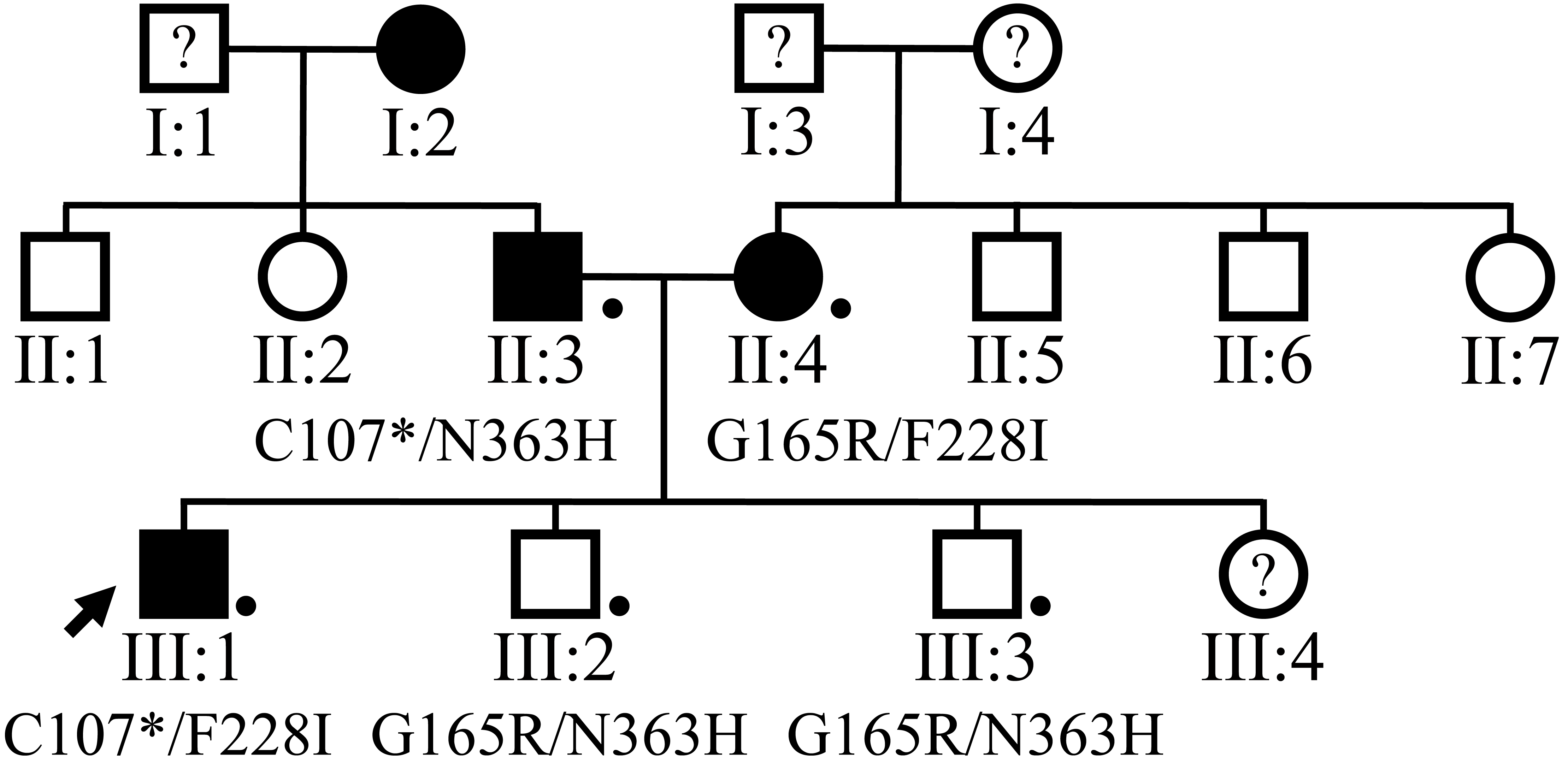** |
| --- | --- |


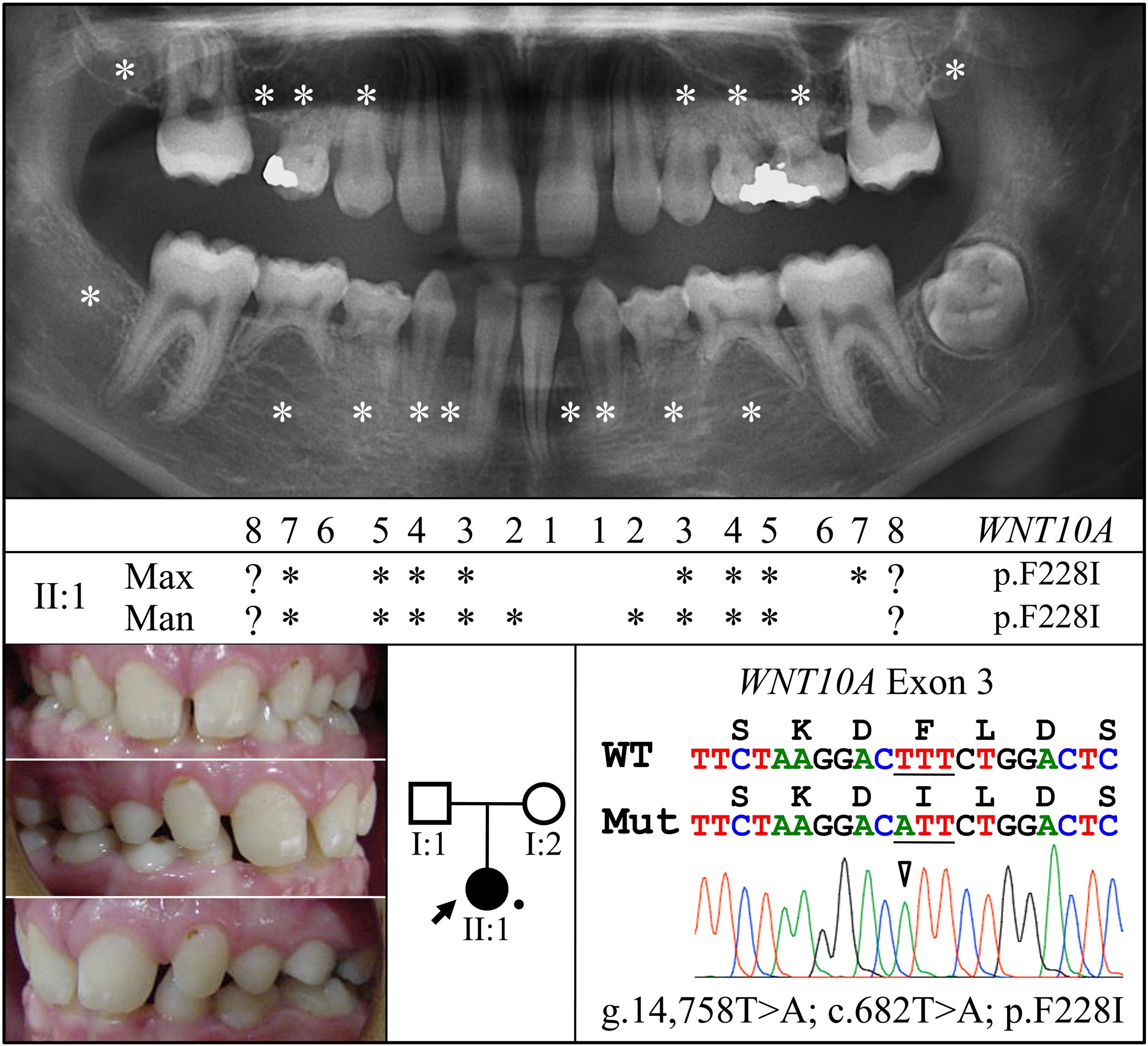


**Figure S15.** Radiograph (age 10y6mo), chart, oral photographs (age 11y), pedigree, and sequencing chromatogram of the proband (II:1) in Family 4. ***Key:*** *, tooth never formed; ?, unknown if tooth will form because of the subjects age at the time of the radiograph. The chromatogram of *WNT10A* exon 3 sequence in exon 3 shows the proband was homozygous for the p.Phe228Ile variation (arrowhead). The genomic and c.DNA descriptions of the sequence variation are numbered with respect to the first nucleotide of the NCBI *WNT10A* genomic reference sequence (NG_012179.1) and the first nucleotide of the *WNT10A* translation initiation codon in the NCBI *WNT10A* mRNA reference sequence NM_025216.2. No other *WNT10A* sequence variations were observed.


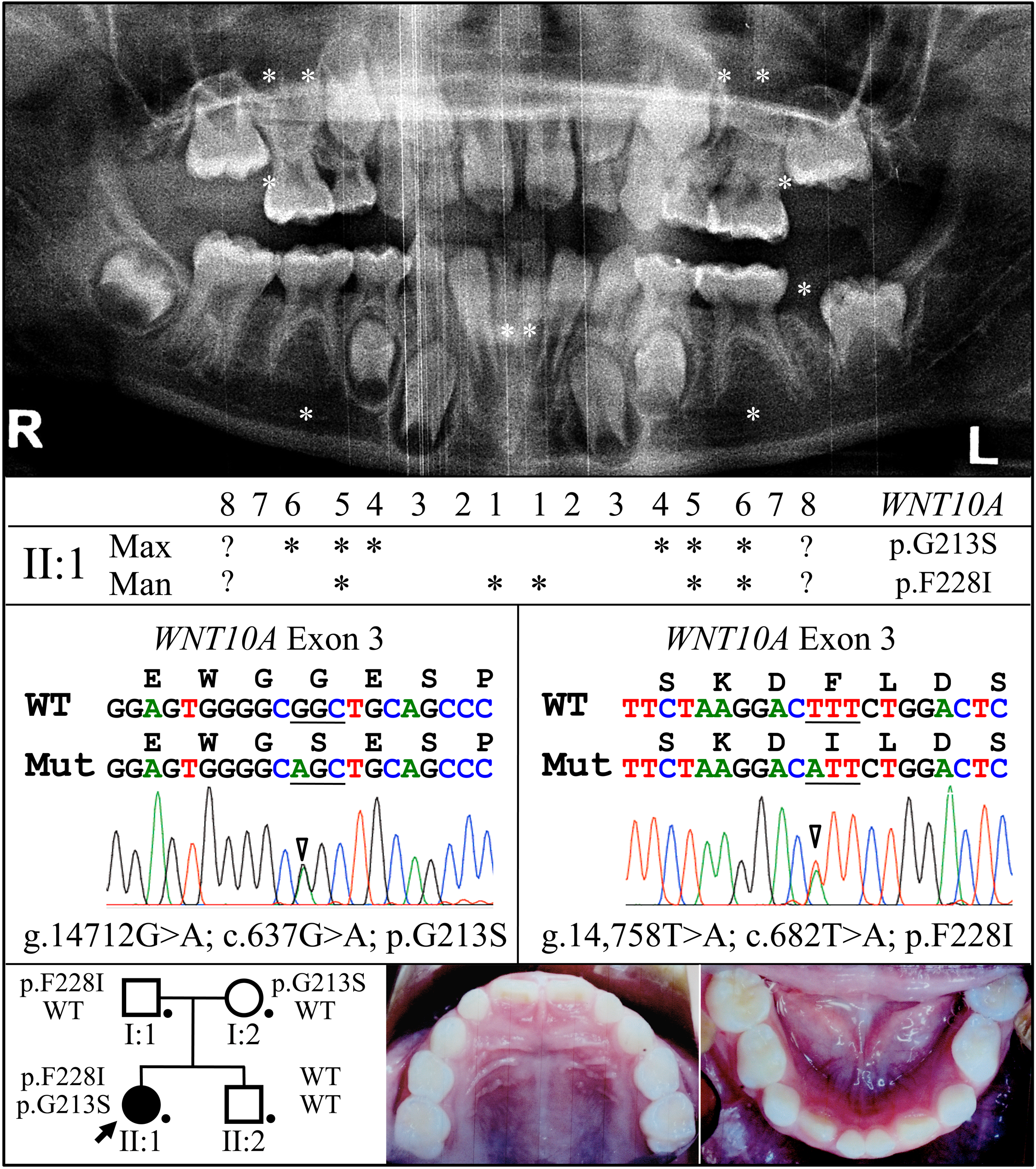


**Figure S16.** Radiograph, chart, sequencing chromatograms, pedigree, and oral photographs of subject (II-1), the proband of Family 5. ***Key:*** *, tooth never formed; ?, unknown if tooth will form because of the subjects age at the time of the radiograph. The *WNT10A* exon 3 chromatograms show that the proband was heterozygous for the p.Gly213Ser and p.Phe228Ile sequence variation. The descriptions of the sequence variations are numbered with respect to the NCBI *WNT10A* genomic (NG_012179.1) and mRNA (NM_025216.2) reference sequences (NM_025216.2). No other *WNT10A* sequence variations were observed.


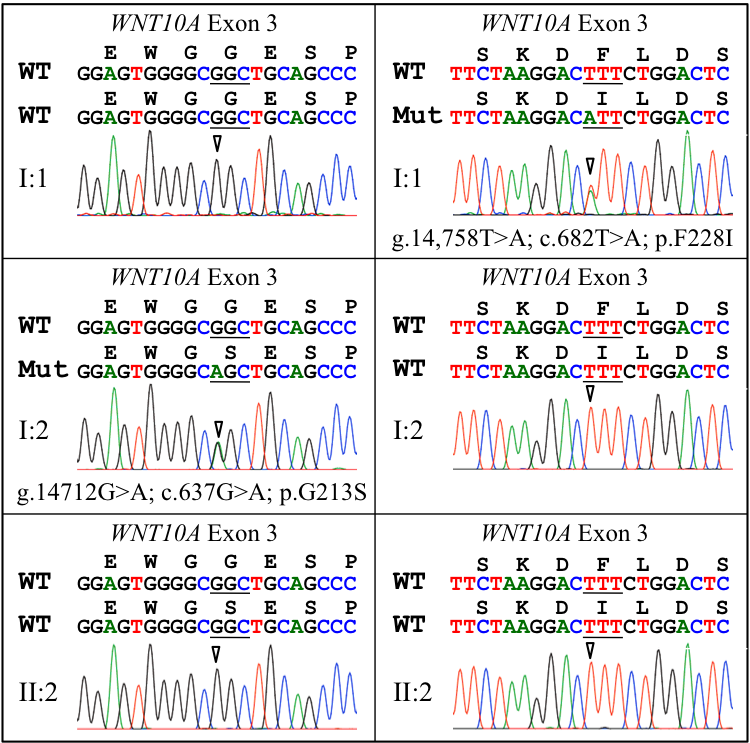


| **Figure S17.** Chromatograms of Family 5 members I:1, I:2, and II:2. The proband’s unaffected father (I:1) was heterozygous for the *WNT10A* p.Phe228Ile mutation. The proband’s unaffected mother (I:2) was heterozygous for the *WNT10A* p.Gly213Ser mutation. The proband’s was compound heterozygous for these *WNT10A* mutations and was missing 11 teeth excluding third molars. | **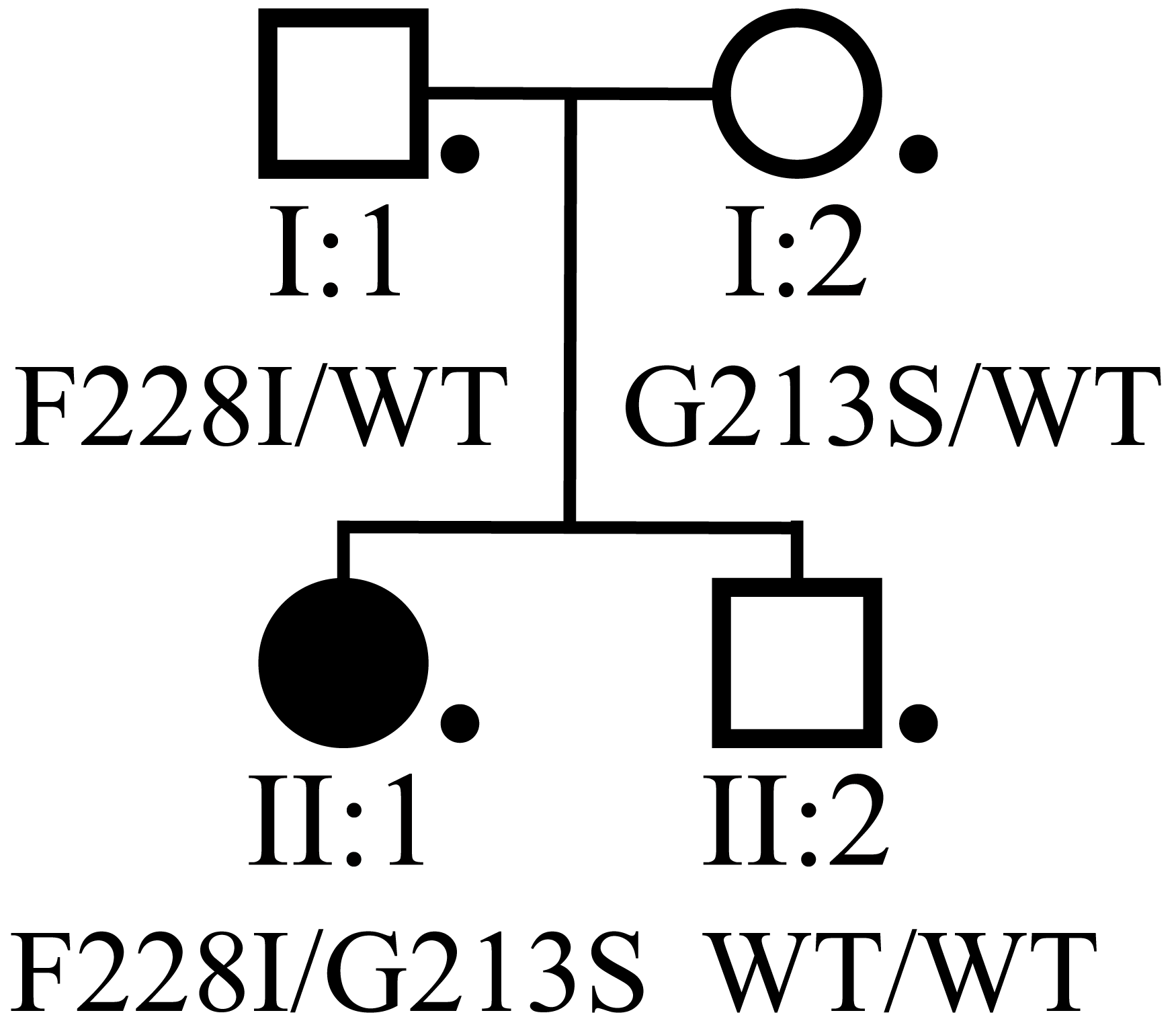** |
| --- | --- |


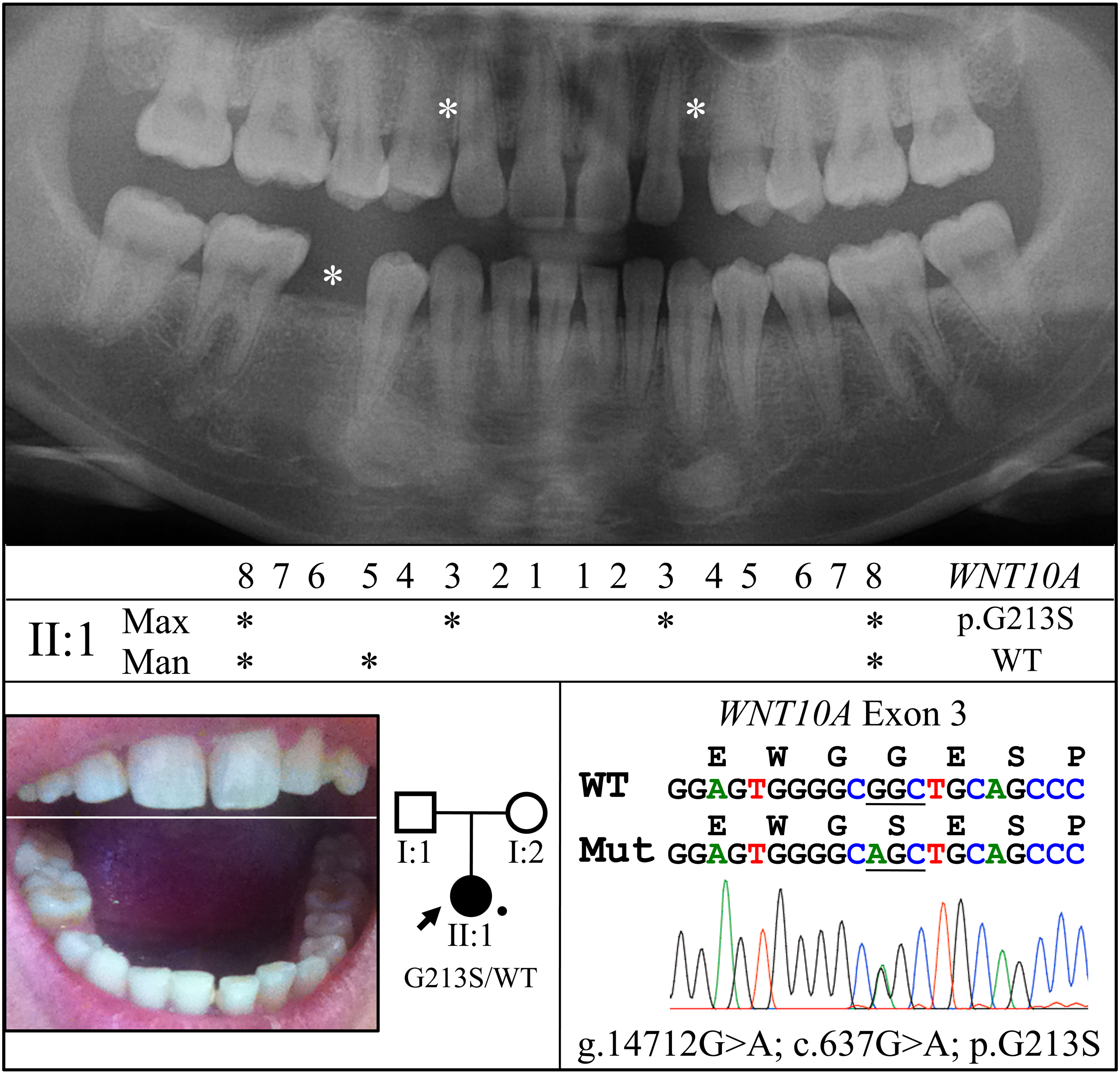


**Figure S18.** Radiograph, chart, oral photograph, pedigree and sequencing chromatogram of subject (II-1), the proband of Family 6. ***Key:*** *, tooth never formed. Radiograph and photograph were taken at age 28. The *WNT10A* exon 3 chromatogram shows that the proband was heterozygous for the p.Gly213Ser sequence variation. The descriptions of the sequence variations are numbered with respect to the NCBI *WNT10A* genomic (NG_012179.1) and mRNA (NM_025216.2) reference sequences (NM_025216.2). No other *WNT10A* sequence variations were observed.
